# Burden and trends of chronic kidney disease due to type 2 diabetes mellitus in China and G20 countries, 1990–2023: a comparative analysis

**DOI:** 10.3389/fendo.2026.1853478

**Published:** 2026-06-10

**Authors:** Jincheng Li, Xinyu Li, Hongtao Zhao, Xinyu Yang, Yulin Li

**Affiliations:** 1 School of Medical and Life Sciences, Chengdu University of Traditional Chinese Medicine, Chengdu, China; 2 State Key Laboratory of Biotherapy, Sichuan University, Chengdu, China

**Keywords:** China, decomposition analysis, diabetic kidney disease, G20 countries, GBD, health inequality

## Abstract

**Background:**

Using the 2023 Global Burden of Disease (GBD) database, we systematically compared the burden of chronic kidney disease due to type 2 diabetes mellitus (T2DM-CKD) between China and G20 countries from 1990 to 2023, and projected trends in the disease burden over the next 26 years.

**Methods:**

We used the Joinpoint model to analyze trends in the disease burden over time, applied the Das Gupta decomposition method, and utilized the concentration index and the slope inequality index to assess health inequalities. Finally, we employed ARIMA (Auto Regressive Integrated Moving Average) and BAPC (Bayesian Age-Period-Cohort) models to forecast trends for the period 2024–2050.

**Results:**

Over the past 33 years, China’s age-standardized incidence, death, and DALY rates (ASIR, ASDR, ASDALYR) all showed a downward trend, with the average annual percentage change (AAPC) of –0.72%, –2.02%, and –1.45%, but aging has driven a steady rise in the absolute burden. The burden of ASIR is concentrated in low Socio-demographic Index (SDI) countries (Concentration Index (CI): –0.168 to –0.277), while the burden of ASDR is shifting toward high-SDI countries (CI: –0.312 to –0.161). The BAPC model projected a 23.2% increase in age-standardized incidence to 2050 (the ARIMA model yielded an unrealistic over-estimation and is not emphasized).

**Conclusion:**

China must prepare for a future with a growing population living with chronic conditions and focus on reducing incidence rates among the elderly. Meanwhile, G20 countries require tailored interventions and joint efforts to reduce health inequalities across SDI levels.

## 1 Background

Chronic kidney disease (CKD) is a common and serious complication of type 2 diabetes mellitus (T2DM). According to GBD 2023 study, the global prevalence of CKD reached 788 million in 2023—a 108% increase from 1990—with an age-standardized prevalence of 14.2% (1). Type 2 diabetes is the leading cause of CKD, and about 27% of people with T2DM develop the condition (2). Rising global obesity and diabetes prevalence are expected to further increase the burden of T2DM-CKD (3). The burden of T2DM-CKD in China is particularly severe; In 2021, China had over 20.91 million diabetic kidney disease cases, with an age-standardized prevalence of 1, 053.92 per 100, 000 person-years (4). The number of adults with CKD in China is approximately 82 million, with diabetes having become the leading cause (5). Thus, understanding T2DM-CKD trends in China relative to other countries is essential for shaping health policy and guiding clinical practice.

The G20 brings together 20 major economies (6). In our analysis, the G20 sample comprises the 18 economies excluding China and the European Union. This specification ensures the statistical independence of China’s data and precludes the duplication of data from EU member states. As major global economies, the G20 covers over two-thirds of the world’s population and spans the full spectrum of development, from emerging to advanced economies. Thus, the disease burden patterns of G20 countries hold significant implications for global public health policy (7).Previous studies have systematically analyzed the global and regional burden of T2DM-CKD (8), but comparative studies specifically focusing on G20 countries remain relatively limited (4). Previous studies show that diabetic kidney disease prevention and control vary significantly across countries at different economic development levels: ASIR peaked in high-SDI countries (23.07 per 100, 000), while ASDR remained well controlled; countries with medium SDI, however, face the pressure of rapidly rising age-standardized incidence rates, with decomposition analysis showing that population growth and aging account for 80% of the increase in age-standardized incidence (9). These differences suggest that a systematic comparison of the burden of T2DM-CKD and its driving factors across G20 countries is of great significance for identifying successful prevention and control strategies to reduce global health inequalities.

Based on the GBD 2023 database, we systematically analyzed trends in the disease burden of T2DM-CKD in China and G20 countries from 1990 to 2023. Our findings offer evidence to inform T2DM-CKD prevention and control in China, and provide policy insights for diabetic kidney disease management globally, especially in middle-income countries.

## 2 Materials and methods

### 2.1 Data sources

The data for this study were obtained from the GBD 2023 database, published by the Institute for Health Metrics and Evaluation (IHME). All data were downloaded using the GHDx query tool. Multi-source data in GBD 2023 were integrated and modeled using the DisMod-MR 2.1 Bayesian meta-regression framework, providing estimates of incidence, prevalence, death, and DALYs for 375 diseases across 204 countries and regions from 1990 to 2023 (1). Since the data used in this study are all publicly available, de-identified, aggregated data, no ethical review approval was required.

In this study, interval estimates were derived using two distinct approaches corresponding to the statistical frameworks employed. For the Bayesian Age–Period–Cohort (BAPC) projections, 95% uncertainty intervals (UI) were calculated as the 2.5th and 97.5th percentiles of the posterior distributions, thereby capturing multiple sources of uncertainty (e.g., parameter, model, and data). In contrast, for all other analyses—including GBD-derived estimates, Joinpoint regression, decomposition analysis, inequality indices, and ARIMA forecasts—95% confidence intervals (CI) were computed based on classical frequentist statistics, where the interval is defined as the point estimate ± 1.96 × standard error (under the assumption of approximate normality).

### 2.2 Study sample and definitions

In this study, the ‘G20’ aggregate excludes China to ensure statistical independence. Furthermore, to prevent double counting, we applied a strict mutual exclusivity principle to EU data, utilizing either EU-level aggregates or individual member state data within each analytical dimension.

### 2.3 Definition of the disease

According to the GBD 2023 classification criteria, chronic kidney disease due to type 2 diabetes mellitus (T2DM-CKD) is defined as structural or functional abnormalities of the kidneys caused by type 2 diabetes, lasting for more than 3 months, primarily manifested by an albumin-to-creatinine ratio ≥ 30 mg/g and/or an estimated glomerular filtration rate < 60 mL/min per 1.73 m² (ICD-10 codes E11.2–E11.29) (10). It should be noted that this disease code in the GBD database does not include chronic kidney disease caused by type 1 diabetes or other causes, and the GBD team has implemented standardized procedures to harmonize data across countries and time periods (1). The disease burden indicators analyzed in this study mainly include: number of incident cases, number of deaths, DALYs, and the corresponding ASIR, ASDR, and ASDALYR.

### 2.4 Descriptive analysis

ASIR, ASDR, and ASDALYR were used to estimate the burden of T2DM-CKD. Age-standardized rates (ASR) were calculated using the direct method with the GBD global standard population as the reference, expressed per 100, 000 population. The EAPC was calculated using the linear regression model Y = α + βX + ϵ, where Y = ln (ASR), X = year, and ϵ is the error term. The EAPC was then calculated as 100 × [exp (β) − 1], corresponding to the annual percentage change in ASR. The 95% confidence interval (CI) for the EAPC was derived from the linear regression model. An upward trend was considered when both the EAPC and the lower bound of its 95% CI were positive. A downward trend was considered when both the EAPC and the upper bound of its 95% CI were negative. Otherwise, the trend was considered stable.

We used bivariate visualizations (*ggplot2 in R*) to examine disease burden patterns by sex, age, and year. Furthermore, age-specific trends in incidence, death, DALYs, prevalence, YLDs, and YLLs for T2DM-CKD were systematically compared between China and G20 countries, with data stratified by 5-year age groups and visualized using line charts.

### 2.5 Joinpoint regression model

We analyzed trends in ASIR, ASDR, and ASDALYR in China and G20 countries from 1990 to 2023 using Joinpoint regression (version 5.2.0). The analysis allowed for a maximum of five turning points. Grid search combined with the Monte Carlo permutation test was used to identify the optimal number and location of turning points, with a significance level set at α = 0.05. We used natural logarithmic transformation for each time period to estimate the APC and its 95% confidence interval, along with the AAPC for the whole study period. An APC below zero indicates a downward trend, while an APC above zero indicates an upward trend. The AAPC represents the average trend over 1990–2023. If no significant inflection points are detected, the APC equals the AAPC; otherwise, the APC varies across periods, and the AAPC summarizes the overall change.

### 2.6 Decomposition analysis

To quantify the contribution of demographic and epidemiological factors to the absolute change in T2DM-CKD cases, we employed the Das Gupta decomposition method. This approach partitions the total absolute increase into three distinct components: population growth, population aging, and changes in epidemiological rates (11). Compared with alternative decomposition techniques, the Das Gupta method offers a mathematically complete and symmetric solution without residual interaction terms, and it has been widely adopted in GBD studies (12).

### 2.7 Analysis of health inequalities

To quantify how socioeconomic development relates to disease burden, we calculated the SII and CI for 1990 and 2023 following the GBD study framework (1). We calculated the slope index of inequality (SII) using weighted linear regression of ASIR, ASDR, and ASDALYR against relative SDI rank. The SII quantifies absolute disparities in disease burden across the SDI gradient. A positive SII means a heavier burden in high-SDI countries, whereas a negative SII means a heavier burden in low-SDI countries. The concentration index (CI) ranges from –1 to 1, where positive values indicate a greater burden in high-SDI countries, negative values indicate a greater burden in low-SDI countries, and zero indicates perfect equality. The CI is derived from the Lorenz curve and measures how concentrated the disease burden is across SDI levels (13). All analyses were conducted using R software (Version 4.4.1).

Based on the average annual percentage change (AAPC) in ASIR, ASDR, and ASDALYR, G20 countries were classified into four trend categories. Detailed classification criteria and results are presented in **Supplementary Figure 1**.

### 2.8 Predictive analytics

#### 2.8.1 Bayesian age-period-cohort model

The Bayesian age-period-cohort (BAPC) model was used to predict trends in ASIR, ASDR, and ASDALYR across different age groups in China and G20 countries from 2024 to 2050. We fitted the model using age-specific data (5-year age groups) from 1990 to 2023, incorporating age, period, and cohort effects. Posterior estimates were obtained using the Integrated Nested Laplace Approximation (INLA) algorithm with default weakly informative priors. Model adequacy was assessed using the deviance information criterion (DIC); the default second-order random walk (RW2) prior was retained as it yielded stable projections consistent with previous studies (14, 15). Since negative lower bounds of the prediction intervals are not plausible in an epidemiological context, they were truncated at zero. The point estimates and upper bounds remain unchanged.

#### 2.8.2 Auto regressive integrated moving average model

The ARIMA model was used for forecasting (16, 17) to complement the results of the BAPC model. First, a stationarity test (Augmented Dickey-Fuller test) was performed on the age-standardized rate series. If the series was non-stationary, it was made stationary through differencing. The *auto.arima* function in the *forecast* package of *R software* was used to automatically select the optimal model parameters (p, d, q) based on the Akaike Information Criterion (AIC). After model fitting, the Ljung-Box test was used to verify that the residuals were white noise. Based on the selected optimal model, point forecasts and 95% confidence intervals for the period 2024–2050 were calculated. All ARIMA analyses were performed using R software (version 4.4.1).

## 3 Results

### 3.1 Overview of the disease burden of T2DM-CKD and its age and sex distribution.

#### 3.1.1 Overall trends

**Figure 1:**
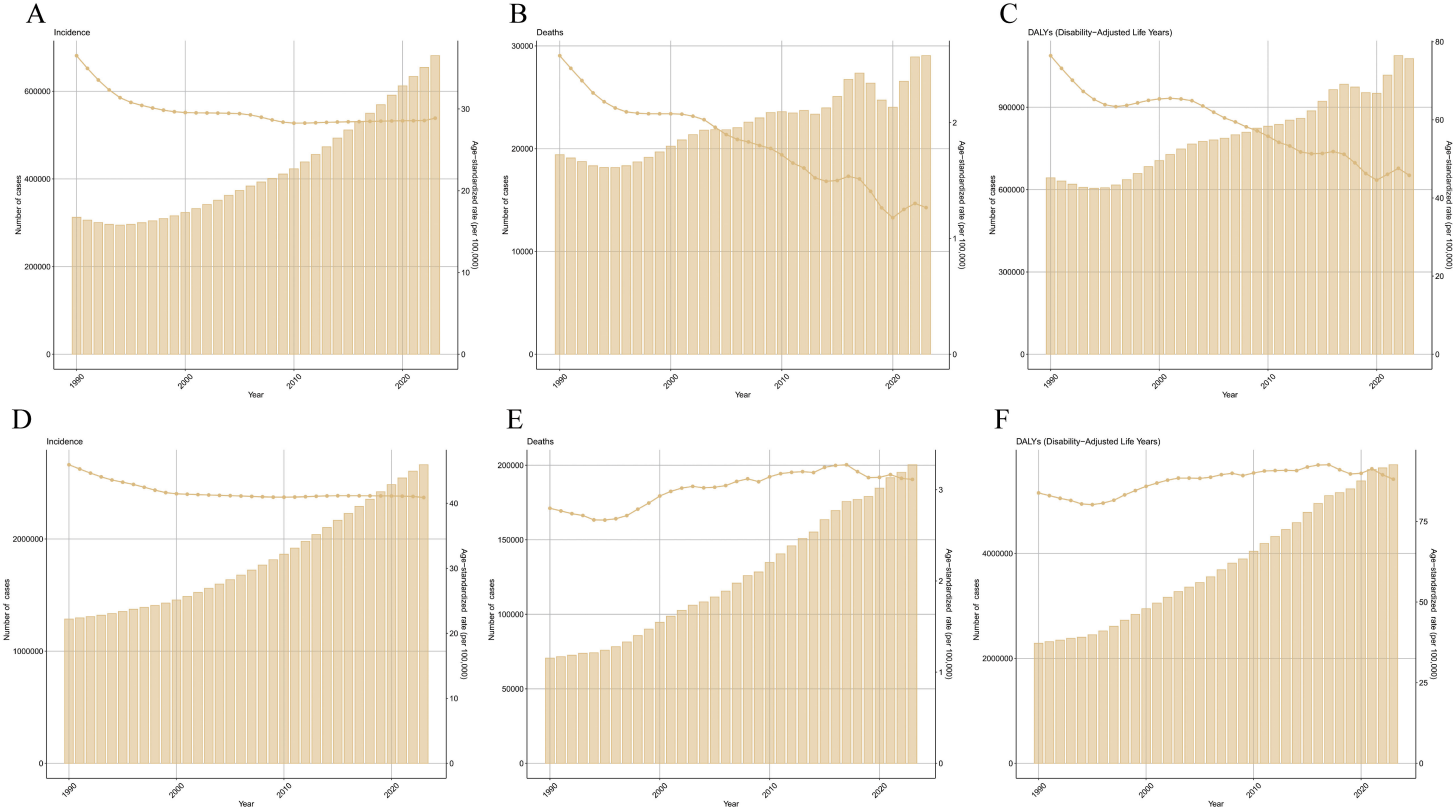
Trends in the number of cases and ASIR, ASDR, ASDALYR for T2DM-CKD in China and G20 countries, 1990–2023. **(A)** China, incidence;**(B)** China, death;**(C)** China, DALYs;**(D)** G20 countries, incidence; **(E)** G20 countries, death; **(F)** G20 countries, DALYs. G20 in this analysis excludes China and the European Union to avoid double-counting.

**Figure 1**; **Table 1** illustrate the trends in the number of cases and ASIR, ASDR, ASDALYR for T2DM-CKD in China and G20 countries from 1990 to 2023. In 2023, the incident cases with T2DM-CKD in China were 681, 203.38 (95% CI: 620, 941.41–763, 650.75), including 340, 532.82 male patients (95% CI: 310, 923.56–379, 719.88) and 340, 670.56 female patients (95% CI: 310, 361.53–383, 191.18). ASIR was 28.87 per 100, 000 population (95% CI: 26.57–32.34), ASDR was 1.27 per 100, 000 population (95% CI: 0.87–1.68), and ASDALYR was 45.80 per 100, 000 population (95% CI: 35.78–56.63). Compared with 1990, DALYs increased from 642, 711.78 (95% CI: 472, 563.30–891, 340.59) to 1, 077, 141.47 (95% CI: 839, 438.59–1, 332, 417.87), representing an increase of 67.59%. Between 1990 and 2023, the ASIR, ASDR, and ASDALYR all showed a downward trend (AAPC: –0.72%, –2.02%, –1.45%), but the absolute burden increased. During the same period, ASIR, ASDR, and ASDALYR in G20 countries were all significantly higher than those in China.

**Table 1:** ASR and EAPC in T2DM-CKD incidence, deaths, and DALYs across G20 countries from 1990 to 2023.

Country/Region	Sex	incidence			Death			DALYs		
		ASR-1990	ASR-2023	EAPC(95%CI)	ASR-1990	ASR-2023	EAPC(95%CI)	ASR-1990	ASR-2023	EAPC(95%CI)
G20	Both	45.94 (41.74-51.55)	40.9 (37.6-45.72)	0.57 (0.36-0.78)	2.8 (2.15-3.59)	3.11 (2.49-3.69)	1.54 (1.3-1.79)	83.84 (67.09-101.39)	88.1 (73.52-104.45)	1.23 (1.02-1.45)
	female	44.23 (40.13-49.71)	39.34 (36.03-43.99)	-0.25 (-0.31--0.19)	2.32 (1.72-3)	2.76 (2.17-3.41)	0.74 (0.62-0.87)	70.07 (55.98-85.49)	77.29 (63.67-94.13)	0.5 (0.41-0.6)
	male	48.18 (43.89-53.96)	42.65 (39.31-47.5)	-0.26 (-0.34--0.19)	3.47 (2.57-4.58)	3.54 (2.8-4.4)	0.36 (0.26-0.45)	101.03 (79.16-128.87)	100.25 (83.65-118.79)	0.23 (0.16-0.31)
Argentina	Both	26.15 (23.6-29.13)	26.55 (24.45-28.92)	0.47 (0.32-0.63)	4.99 (3.7-6.32)	4.41 (3.17-5.65)	-0.14 (-0.4-0.11)	103.16 (76.95-129.87)	90.44 (68.22-112.63)	-0.23 (-0.44--0.01)
Australia	Both	40.79 (36.56-44.78)	36.8 (33.06-41.09)	0.33 (-0.14-0.8)	0.91 (0.66-1.24)	2.79 (2.35-3.16)	6.87 (6-7.75)	25.95 (19.94-32.75)	57.06 (49.92-64.39)	4.55 (3.88-5.23)
Brazil	Both	46.84 (41.86-53.37)	46.15 (41.52-52.31)	1.24 (0.91-1.57)	3.88 (3.1-4.66)	4.29 (3.32-5.28)	1.51 (1.05-1.97)	113.9 (93.46-136.15)	115.29 (93.95-138.54)	1.15 (0.77-1.52)
Canada	Both	35.76 (31.47-42.57)	32.31 (29.24-35.92)	0.64 (0.08-1.21)	1.29 (0.96-1.65)	2.43 (2.06-2.85)	3.55 (2.84-4.27)	37.15 (29.71-45.8)	54.47 (46.89-62.74)	2.61 (1.99-3.23)
China	Both	36.52 (32.98-41.15)	28.87 (26.57-32.34)	0.8 (0.46-1.14)	2.58 (1.64-3.93)	1.27 (0.87-1.68)	-0.66 (-1.02--0.3)	76.43 (56.46-107.02)	45.8 (35.78-56.63)	-0.11 (-0.44-0.22)
France	Both	25.84 (23.84-28.37)	27.41 (24.94-30.62)	0.66 (-0.02-1.35)	0.8 (0.58-1.09)	0.91 (0.64-1.27)	1.43 (0.41-2.47)	23.79 (18.66-29.63)	24.88 (19.37-30.53)	0.75 (-0.06-1.57)
Germany	Both	40.84 (36.93-45.16)	42.29 (39.65-45.1)	0.69 (-0.13-1.51)	1.27 (0.92-1.66)	2.02 (1.37-2.72)	3.2 (2.1-4.3)	37.78 (30.4-46.23)	42.47 (33.15-52.96)	1.61 (0.67-2.56)
India	Both	54.96 (49.84-61.71)	43.67 (39.85-49.1)	0.17 (-0.35-0.69)	3.57 (2.49-4.74)	2.44 (1.71-3.29)	-0.32 (-0.98-0.34)	118.65 (92.41-147.63)	92.46 (73.14-115.6)	0.14 (-0.39-0.67)
Indonesia	Both	39.52 (36.26-43.89)	43.7 (40.33-48.29)	1.13 (0.63-1.63)	6.74 (4.18-10.05)	6.85 (4.49-10.08)	1.2 (0.59-1.8)	184.22 (124.88-260.17)	201.99 (144.05-277.54)	1.46 (0.98-1.94)
Italy	Both	33.65 (30.58-37.68)	33.04 (30.01-36.96)	0.59 (-0.28-1.46)	1.06 (0.77-1.41)	1.09 (0.76-1.51)	1.27 (0.15-2.4)	32.34 (25.57-40.93)	33.79 (25.57-41.56)	0.83 (-0.11-1.78)
Japan	Both	69.51 (63.34-77.23)	70.73 (64.59-78.51)	0.75 (-0.1-1.6)	4.64 (3.67-5.67)	2.95 (2.24-3.81)	0.72 (-0.5-1.95)	108.98 (89.4-127.91)	77.59 (61.28-93.99)	0.42 (-0.6-1.45)
Mexico	Both	85.9 (77.51-95.62)	90.22 (81.72-100.01)	1.35 (0.88-1.83)	6.55 (5.11-8.06)	9.38 (7.19-11.39)	2.92 (2.12-3.74)	195.77 (161.66-236.6)	273.88 (218.66-331.57)	2.69 (2-3.38)
Republic of Korea	Both	54.77 (49.12-61.22)	40.31 (36.91-43.52)	0.94 (0.5-1.39)	3.93 (2.74-6.57)	3.83 (2.2-5.33)	2.01 (1.49-2.53)	94.05 (70.36-139.34)	86.05 (61.88-108.43)	1.67 (1.21-2.13)
Russian Federation	Both	41.41 (37.38-46.73)	42.63 (38.67-48.01)	0.62 (0.16-1.08)	0.32 (0.23-0.43)	0.55 (0.39-0.73)	2.69 (2.11-3.27)	22.12 (16.68-28.84)	22.79 (17.76-29.04)	0.59 (0.11-1.07)
Saudi Arabia	Both	45.77 (41.31-51.1)	48.47 (43.87-53.87)	0.72 (-0.25-1.71)	10.55 (6.65-15.09)	12.61 (7.91-18.49)	1.32 (-0.21-2.89)	230.54 (153.78-329.89)	254.81 (167.17-352.4)	1.15 (-0.11-2.43)
South Africa	Both	70.27 (64.19-77.82)	81.16 (74.12-89.21)	0.92 (0.4-1.44)	2.67 (1.75-3.72)	3.73 (2.52-5.26)	2.28 (1.6-2.97)	84.02 (61.29-110.23)	110.25 (83.4-143.74)	1.8 (1.22-2.39)
Turkey	Both	45.82 (41.38-50.95)	45 (40.57-49.9)	1.09 (0.75-1.43)	7.12 (4.19-10.54)	4.83 (3.23-6.8)	0.03 (-0.51-0.57)	160.48 (104.16-236.76)	108.91 (77.9-140.77)	-0.1 (-0.52-0.32)
United Kingdom	Both	52.53 (47.67-58.81)	42.56 (38.66-47.91)	-0.09 (-0.74-0.56)	0.59 (0.44-0.78)	0.95 (0.68-1.24)	2.58 (1.66-3.52)	31.39 (23.7-40.03)	33.98 (25.69-41.86)	1.08 (0.36-1.8)
United States of America	Both	51.2 (46.55-57.75)	51.15 (46.68-57.5)	0.59 (0.14-1.05)	2.13 (1.67-2.6)	8.42 (7.02-9.76)	5.36 (4.77-5.95)	70.41 (58.87-81.75)	193.01 (166.9-218.62)	4.14 (3.64-4.64)

The “G20” excludes both China (to allow for separate analysis) and the European Union (to prevent duplication of member states).

#### 3.1.2 Age distribution

**Figure 2:**
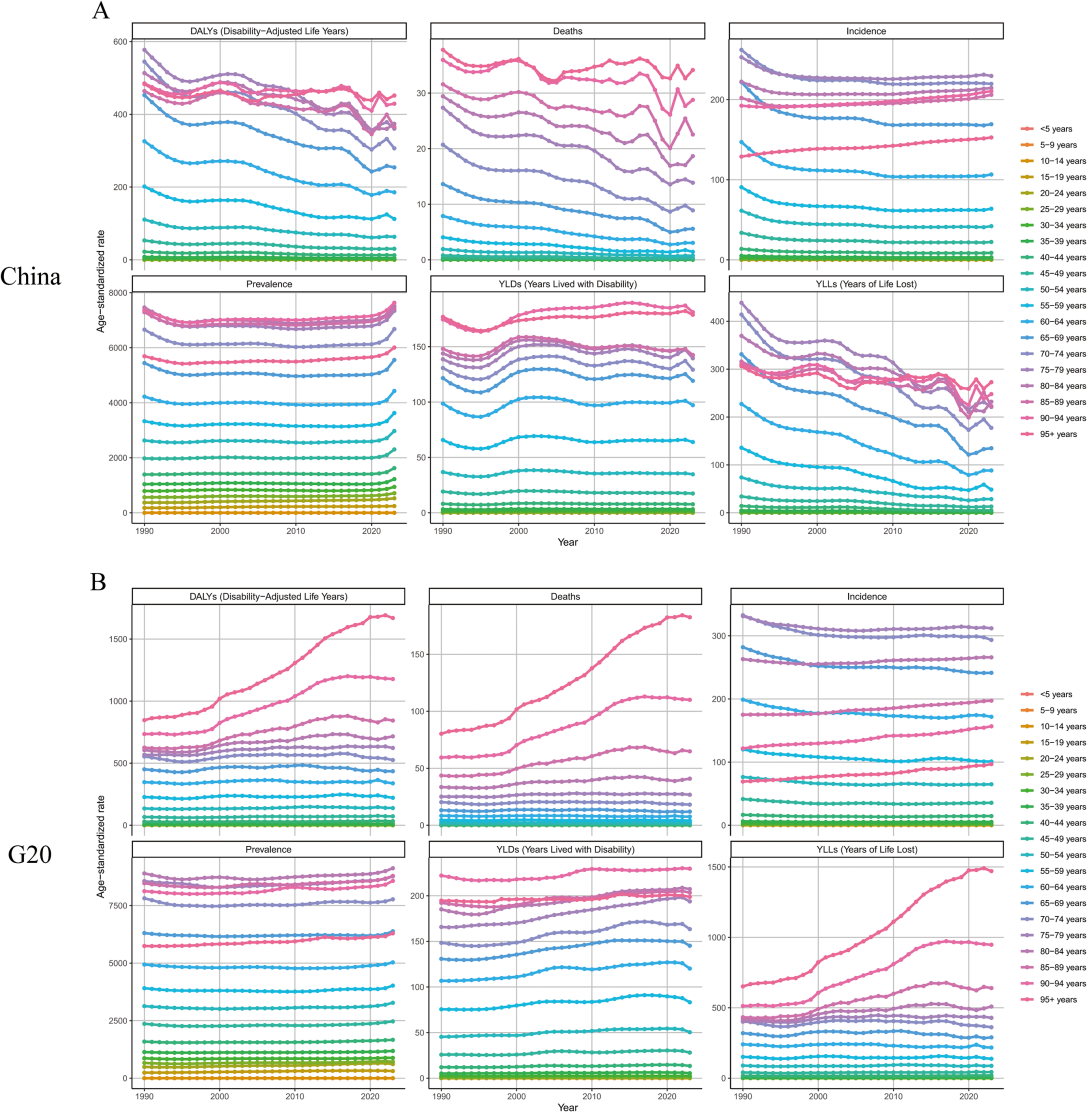
Age−specific trends in DALYs, death, incidence, prevalence, YLDs and YLLs for T2DM−CKD in China and G20 countries, 1990–2023. **(A)** China; **(B)** G20 countries. G20 in this analysis excludes China and the European Union to avoid double-counting.

**Figure 2** illustrates the trends in age-specific rates of DALYs, incidence, death, prevalence, YLDs, and YLLs for T2DM-CKD across different age groups. Cases were most frequent in people aged 60 and above. For DALYs, the peak ASDALYR in China shifted from the 75–79 age group before 2010 to the 95+ age group after 2010, whereas in G20 countries, the peak ASDALYR has consistently been in the 95+ age group and increased steadily with age. ASDR also exhibited an increasing trend with age, consistent with the trends in ASDALYR.

**Figure 3:**
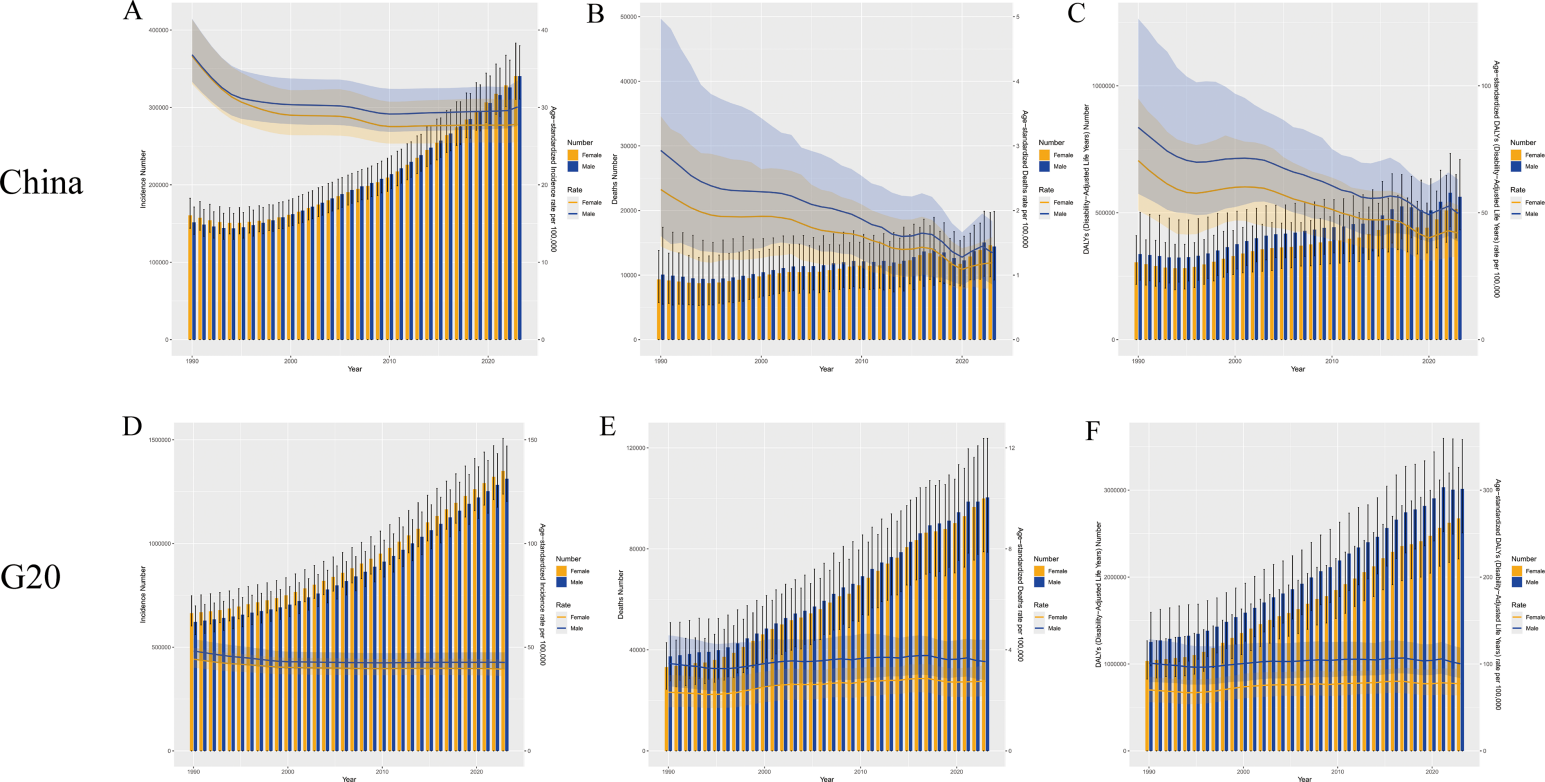
Changes in the number of cases and ASIR, ASDR and ASDALYR by sex during the study period (1990–2023). **(A)** China, incidence;**(B)** China, death;**(C)** China, DALYs;**(D)** G20 countries, incidence; **(E)** G20 countries, death; **(F)** G20 countries, DALYs. G20 in this analysis excludes China and the European Union to avoid double-counting.

**Figure 3** shows the changes in age-standardized rates (ASR) and the number of cases of T2DM-CKD by sex in China and G20 countries from 1990 to 2023. The bar charts show that the number of incident cases, deaths, and DALYs increased over time in both China and G20 countries. However, the age-standardized rates for T2DM-CKD in China generally showed a downward trend, while those in G20 countries remained relatively stable. Furthermore, sex- and age-stratified analysis revealed that age-standardized rates for all indicators were slightly higher in men than in women. Detailed sex- and age-specific trends are presented in **Supplementary Figure 2**.

### 3.2 Regression analysis

**Figure 4:**
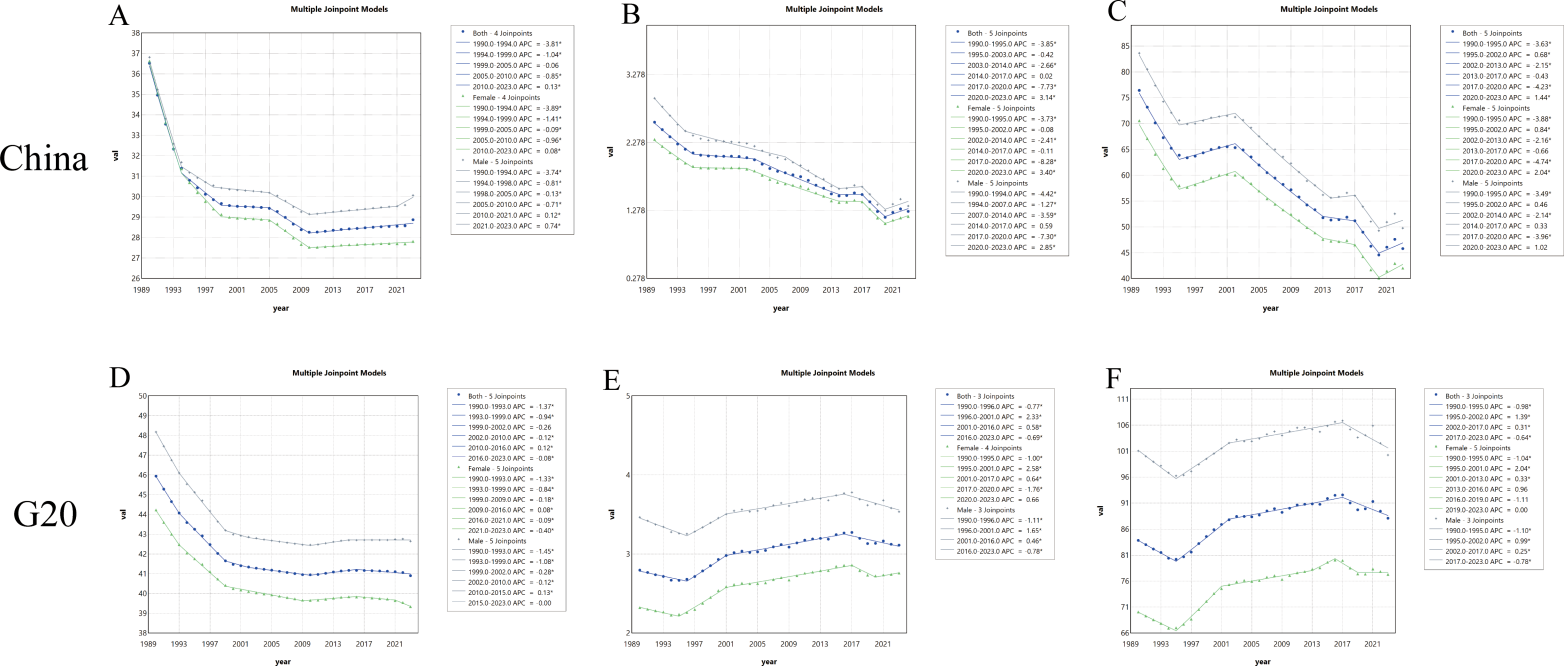
Joinpoint time-trend analysis of ASIR, ASDR and ASDALYR for T2DM‑CKD, 1990–2023 (* indicates P < 0.05). **(A)** China, ASIR; **(B)** China, ASDR; **(C)** China, ASDALYR; **(D)** G20 countries, ASIR; **(E)** G20 countries, ASDR; **(F)** G20 countries, ASDALYR. APC: annual percent change; AAPC: average annual percent change. G20 in this analysis excludes China and the European Union to avoid double-counting.

**Figure 4** shows the time-trend analysis of the relationship between the ASIR, ASDR and ASDALYR of T2DM-CKD in China and G20 countries. From 1990 to 2023, the ASIR, ASDR, and ASDALYR in China all showed a significant overall downward trend, with AAPCs of –0.72% (95% CI: –0.76 to –0.67), –1.45% (95% CI: –1.73 to –1.16), and –2.02% (95% CI: –2.58 to –1.46), respectively. Taking the ASDALYR as an example, it declined rapidly from 1990 to 1995 (APC = –3.63%). From 1995 to 2002, the decline slowed, with a slight increase (APC = 0.68%). Between 2002 and 2013, it resumed a rapid decline (APC = –2.15%), then slowed again from 2013 to 2017 (APC = –0.43%). From 2017 to 2020, it declined rapidly once more (APC = –4.23%), followed by a rebound from 2020 to 2023 (APC = 1.44%). The trend in ASDR generally aligned with that of ASDALYR, although the inflection points and magnitudes of change differed slightly (**Supplementary Tables 1**, **2**).

Between 1990 and 2023, both ASDR and ASDALYR in G20 countries showed a slow overall upward trend. Taking the ASDALYR as an example, the ASDALYR declined rapidly from 1990 to 1995 (APC = –0.98%), followed by a sharp increase during 1995–2002 (APC = 1.39%). The rate then rose at a slower pace from 2002 to 2017 (APC = 0.31%), before finally declining again between 2017 and 2023 (APC = –0.64%). The trend in ASDR generally aligned with that of ASDALYR, while the inflection points and magnitudes of change differed slightly (**Supplementary Tables 1**, **2**). For ASIR, the rates in China and G20 countries showed a downward trend, with similar patterns of change. In China, the rate of decline among women (AAPC = –0.82%, 95% CI: –0.85 to –0.80) was consistently higher than that among men (AAPC = –0.61%, 95% CI: –0.63 to –0.58). The ASIR declined rapidly from 1990 to 1994 (APC = –3.81%), then slowed from 1994 to 1999 (APC = –1.04%), and remained stable from 1999 to 2005 (APC = –0.06%); after 1999, the trends for men and women were similar. Subsequently, from 2005 to 2010, the ASIR showed a downward trend (APC = –0.85%), before rising slowly from 2010 to 2023 (APC = 0.13%).

### 3.3 Contributions and drivers

**Figure 5:**
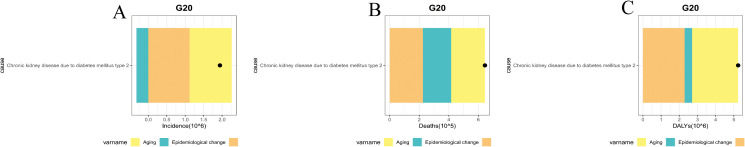
Contribution ratios to the increase in cases, deaths, and DALYs in G20 countries, 1990–2023. The black dots indicate the combined contribution of the three components. **(A)** Incident cases; **(B)** Deaths; **(C)** DALYs. G20 in this analysis excludes China and the European Union to avoid double-counting.

**Figure 5**; **Table 2** present the results of a decomposition analysis of the increases in the number of cases, deaths, and DALYs for T2DM-CKD in G20 countries from 1990 to 2023, attributing this increase to three factors: population growth, population aging, and epidemiological changes. Among the total increase in DALYs, population growth contributed the most, accounting for 48.30%; epidemiological changes (i.e., changes in the disease itself) contributed the second most, at 43.87%; while population aging contributed the least, at only 7.83%. The increase in the number of cases was primarily driven by population aging (58.84%) and population growth (57.66%), while epidemiological changes made a negative contribution (−16.50%), indicating a decline in the inherent risk of the disease. The drivers of the increase in deaths were relatively balanced, with population aging, population growth, and epidemiological changes contributing 35.49%, 34.88%, and 29.63%, respectively.

**Table 2:** Proportion of the increase in the disease burden attributable to three components in China and G20 countries, 1990–2023.

Incidence					Deaths			DALYs		
1941445.76					644602.38			5238706.3		
		Aging (rate)	Population (rate)	Epidemiological change (rate)	Aging (rate)	Population (rate)	Epidemiological change (rate)	Aging (rate)	Population (rate)	Epidemiological change (rate)
G20	Both	1142301.335 (58.84%)	1119532.419 (57.66%)	-320387.99 (-16.50%)	228774.806 (35.49%)	224848.3 (34.88%)	190979.278 (29.63%)	2530440.113 (48.30%)	2298136.95 (43.87%)	410129.234 (7.83%)
	male	597466.884 (61.41%)	553958.554 (56.93%)	-178439.997 (-18.34%)	133433.721 (42.54%)	122794.51 (39.15%)	57415.932 (18.31%)	1454998.744 (54.23%)	1244408.433 (46.38%)	-16438.896 (-0.61%)
	Female	552061.276 (57.02%)	567924.021 (58.65%)	-151728.57 (-15.67%)	102351.603 (31.40%)	107198.4 (32.89%)	116370.901 (35.71%)	1122296.945 (43.95%)	1066819.966 (41.78%)	364327.781 (14.27%)
Mexico	Both	53320.651 (58.74%)	33303.437 (36.69%)	4155.721 (4.58%)	12247.577 (49.15%)	7644.325 (30.68%)	5024.584 (20.17%)	143188.862 (47.92%)	86588.132 (28.98%)	69039.744 (23.10%)
Saudi Arabia	Both	2291.437 (35.89%)	3765.283 (58.97%)	328.378 (5.14%)	1364.579 (31.92%)	2349.78 (54.97%)	560.501 (13.11%)	7220.381 (31.70%)	15161.953 (66.56%)	398.483 (1.75%)
Italy	Both	14936.566 (95.76%)	1381.032 (8.85%)	-720.141 (-4.62%)	2242.781 (49.11%)	192.817 (4.22%)	2131.386 (46.67%)	20793.329 (84.59%)	1458.037 (5.93%)	2329.873 (9.48%)
Turkey	Both	0 (0.00%)	0 (0.00%)	0 (0.00%)	0 (0.00%)	0 (0.00%)	0 (0.00%)	0 (0.00%)	0 (0.00%)	0 (0.00%)
Japan	Both	67607.319 (89.35%)	1482.209 (1.96%)	6572.488 (8.69%)	22429.45 (106.84%)	335.294 (1.60%)	-1771.243 (-8.44%)	251057.897 (152.05%)	2742.079 (1.66%)	-88679.303 (-53.71%)
China	Both	481052.785 (104.31%)	125496.223 (27.21%)	-145388.733 (-31.53%)	61236.153 (207.98%)	17342.063 (58.90%)	-49134.909 (-166.88%)	929864.325 (171.64%)	232552.755 (42.93%)	-620664.995 (-114.57%)
France	Both	8399.612 (56.28%)	4956.701 (33.21%)	1569.609 (10.52%)	1899.143 (56.70%)	626.419 (18.70%)	824.05 (24.60%)	15531.525 (60.34%)	6279.754 (24.40%)	3927.611 (15.26%)
Russian Federation	Both	27209.093 (94.13%)	-2543.647 (-8.80%)	4241.684 (14.67%)	1491.969 (46.88%)	-61.809 (-1.94%)	1752.114 (55.06%)	-18180.028 (455.46%)	-2095.435 (52.50%)	16283.913 (-407.96%)
Australia	Both	5295.587 (48.14%)	7056.914 (64.16%)	-1353.035 (-12.30%)	1564.165 (24.07%)	1797.677 (27.66%)	3137.718 (48.28%)	10609.142 (29.48%)	9833.204 (27.32%)	15548.604 (43.20%)
Germany	Both	22112.115 (73.17%)	3786.415 (12.53%)	4321.25 (14.30%)	3380.235 (23.58%)	608.225 (4.24%)	10346.702 (72.18%)	8096.285 (7.29%)	6217.954 (5.60%)	96737.391 (87.11%)
Korea	Both	0 (0.00%)	0 (0.00%)	0 (0.00%)	0 (0.00%)	0 (0.00%)	0 (0.00%)	0 (0.00%)	0 (0.00%)	0 (0.00%)
United Kingdom	Both	8660.734 (108.66%)	9788.268 (122.81%)	-10478.72 (-131.47%)	663.869 (15.85%)	726.126 (17.34%)	2798.306 (66.81%)	9077.013 (46.19%)	7017.136 (35.71%)	3558.057 (18.11%)
Indonesia	Both	54267.02 (50.35%)	43321.349 (40.19%)	10191.246 (9.46%)	17948.099 (54.00%)	16082.99 (48.39%)	-794.335 (-2.39%)	248683.646 (49.60%)	202942.527 (40.48%)	49745.187 (9.92%)
Brazil	Both	35544.559 (57.40%)	26538.848 (42.86%)	-157.889 (-0.25%)	9827.892 (59.90%)	4728.646 (28.82%)	1851.284 (11.28%)	30847.102 (17.66%)	101342.282 (58.03%)	42454.868 (24.31%)
United States of America	Both	93414.369 (54.39%)	75937.592 (44.22%)	2388.55 (1.39%)	32266.498 (17.29%)	27355.624 (14.66%)	126954.487 (68.04%)	260942.992 (20.83%)	220740.825 (17.62%)	771317.679 (61.56%)
Argentina	Both	3483.59 (37.40%)	5490.172 (58.94%)	341.748 (3.67%)	1718.306 (43.29%)	2833.444 (71.38%)	-582.306 (-14.67%)	16676.775 (54.98%)	21246.986 (70.05%)	-7590.613 (-25.02%)
South Africa	Both	14650.613 (37.77%)	18925.168 (48.80%)	5208.539 (13.43%)	1555.153 (32.02%)	2053.655 (42.29%)	1247.49 (25.69%)	18687.971 (33.57%)	24343.762 (43.73%)	12634.114 (22.70%)
Canada	Both	8831.793 (67.65%)	5984.863 (45.84%)	-1760.701 (-13.49%)	1966.668 (32.00%)	1312.683 (21.36%)	2866.51 (46.64%)	13536.973 (43.24%)	8308.434 (26.54%)	9461.533 (30.22%)
India	Both	228086.313 (59.65%)	283943.235 (74.26%)	-129664.481 (-33.91%)	30157.491 (61.44%)	40911.517 (83.34%)	-21981.304 (-44.78%)	503581.999 (62.25%)	602064.218 (74.42%)	-296626.152 (-36.66%)

The “G20” excludes both China (to allow for separate analysis) and the European Union (to prevent duplication of member states).

**Figure 6:**
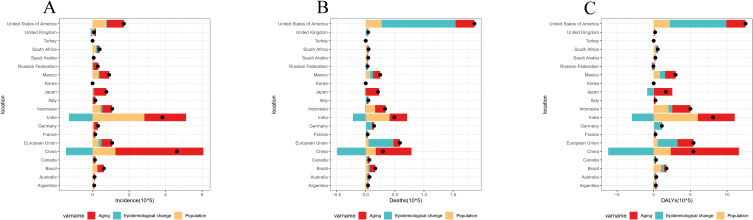
Absolute contributions of individual countries to the overall increase in the disease burden in G20 countries from 1990 to 2023, along with a decomposition analysis of the driving factors for each country. The black dots indicate the combined contribution of the three components. **(A)** Incidence; **(B)** Deaths; **(C)** DALYs. G20 in this analysis excludes China and the European Union to avoid double-counting. China and EU member states are shown as individual economies but are not included in the G20 aggregate.

**Figure 6** shows the absolute contributions of individual countries to the overall increase in the disease burden in G20 countries from 1990 to 2023, along with the results of a decomposition analysis of the driving factors for each country. At the national level, China, India, and the United States were the primary contributors to the increase in the number of cases, accounting for 28.21%, 23.39%, and 10.50% of the total G20 increase, respectively. Notably, the United States accounted for a larger share of the increases in deaths and DALYs, accounting for 39.27% and 27.00% of the total G20 increase, respectively—far exceeding other countries—and epidemiological changes were the primary driving factor.

A decomposition analysis further reveals differences in the driving factors among the three countries. In China, population aging is the primary driver of increases in incidence, deaths, and DALYs, contributing 104.31%, 207.98%, and 171.64%, respectively (the contribution of aging exceeds 100% due to the offsetting effect of epidemiological changes). In India, population growth and aging jointly drive the increase in disease burden. Although epidemiological changes in both China and India contributed negatively, the positive effects of population aging and population growth resulted in total increases that were among the highest in the G20.

### 3.4 Comparison of the disease burden across G20 countries

**Table 1** presents EAPCs in ASIR, ASDR, and ASDALYR for T2DM-CKD in G20 countries from 1990 to 2023. The results show that ASIR, ASDR, and ASDALYR were on an upward trend in most countries. Among them, the United States and Australia had the largest increases in ASDR and ASDALYR, with ASDR values of 5.36 (95% CI: 4.77–5.95) and 6.87 (95% CI: 6.00–7.75), and ASDALYR values of 4.14 (95% CI: 3.64–4.64) and 4.55 (95% CI: 3.88–5.23), respectively. For ASIR, Mexico showed the largest increase (1.35, 95% CI: 0.88–1.83). Meanwhile, for ASDR, only three countries showed a downward trend (Argentina, China, and India), with China experiencing the largest decrease (–0.66, 95% CI: –1.02 to –0.30); for ASDALYR, only three countries showed a downward trend (Argentina, China, and Turkey), with Argentina experiencing the largest decrease (–0.23, 95% CI: –0.44 to –0.01).

**Figure 7:**
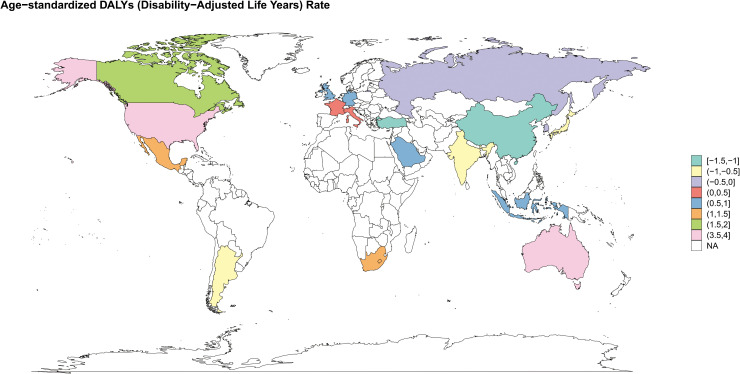
Absolute changes in ASDALYR for T2DM‑CKD in G20 countries, 1990–2023. Map colors represent the change in ASDALYR (ΔASDALYR = ASDALYR_2023 − ASDALYR_1990). Green indicates ΔASDALYR < 0; yellow, orange, and red indicate ΔASDALYR > 0. G20 in this analysis excludes China and the European Union to avoid double-counting. China and EU member states are shown as individual economies but are not included in the G20 aggregate.

**Figure 7** shows the absolute change in ASDALYR for T2DM-CKD in G20 countries from 1990 to 2023 (ΔASDALYR = ASDALYR_2023 − ASDALYR_1990). The United States, Australia, Canada, and Mexico exhibited larger absolute changes, whereas China, India, Japan, and Russia showed smaller absolute changes. Notably, China appears in blue on the map of ASIR (ΔASIR < 0, ΔASIR= ASIR_2023 – ASIR_1990), but its EAPC was positive—suggesting that recent declines in the age-standardized rate have not reversed the long-term upward trend. Detailed map results for ASIR and ASDR are presented in **Supplementary Figure 3**.

### 3.5 Health inequality analysis

**Figure 8:**
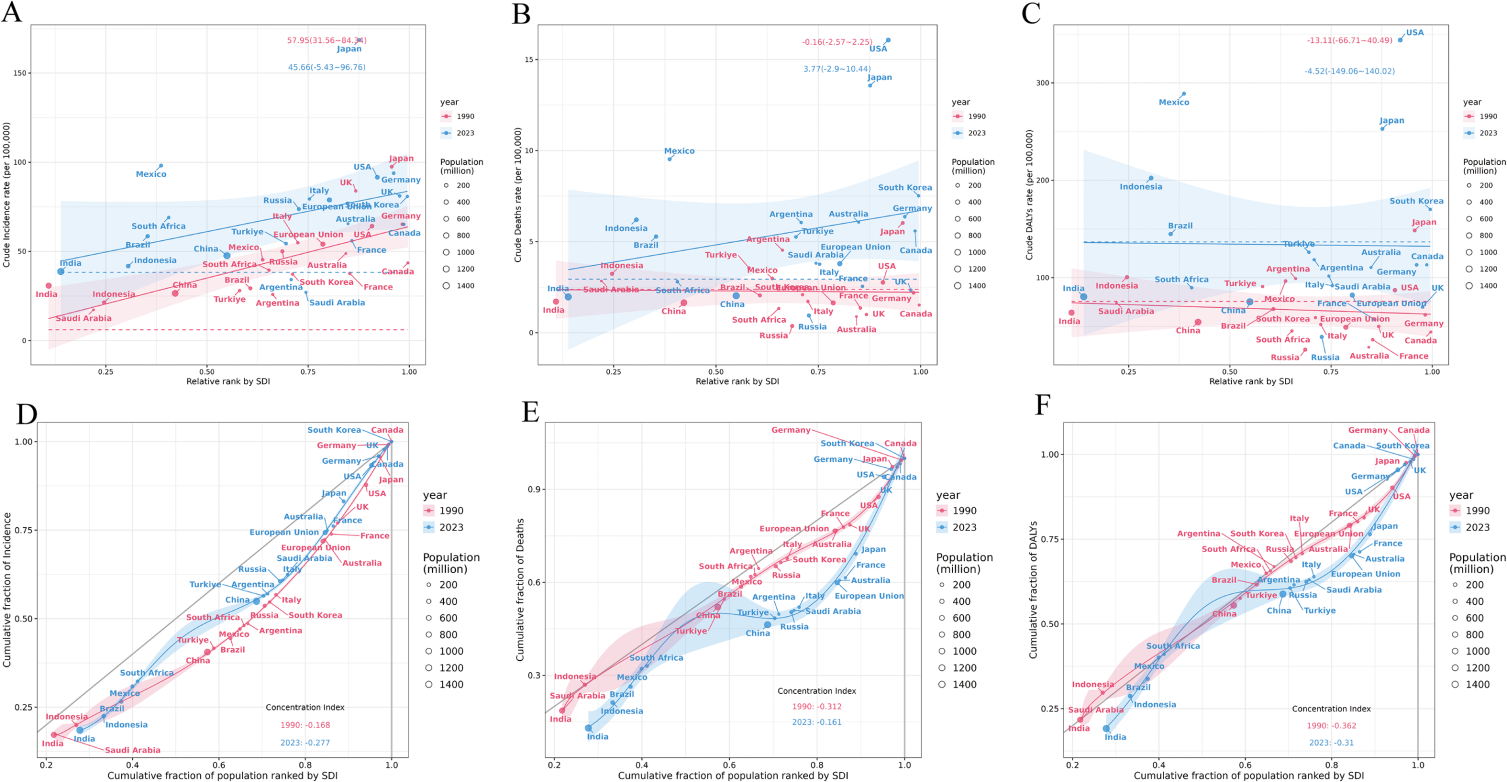
Distribution of ASIR, ASDR and ASDALYR for T2DM‑CKD in G20 countries by SDI in 1990 and 2023, and changes in the concentration index. **(A)** Distribution, incidence; **(B)** Distribution, death; **(C)** Distribution, DALYs; **(D)** Concentration index, incidence; **(E)** Concentration index, death; **(F)** Concentration index, DALYs. G20 in this analysis excludes China and the European Union to avoid double-counting. China and EU member states are shown as individual economies but are not included in the G20 aggregate. The concentration index (CI) ranges from –1 to +1; negative values indicate burden concentrated in lower‑SDI countries, positive values in higher‑SDI countries.

To analyze the relationship between disease burden and socioeconomic development, the distribution of disease burden was further stratified by SDI. **Figure 8** illustrates the distribution of ASIR, ASDR, and ASDALYR for T2DM-CKD in G20 countries in 1990 and 2023 according to SDI, along with changes in the concentration index. The burden of T2DM-CKD in G20 countries exhibits different patterns as SDI changes. ASIR showed a positive correlation with SDI in both years, however, the slope was flatter in 2023, suggesting that the increase was faster in low-SDI countries than in high-SDI countries, and that the incidence burden is gradually shifting toward low-SDI countries. ASDR showed a slight negative correlation with SDI in 1990, whereas this trend reversed to a positive correlation in 2023, indicating that ASDR rose faster in high-SDI countries, and the death burden is concentrating in high-SDI countries. ASDALYR decreased with rising SDI in both years, however, between 1990 and 2023, the increase in DALY rates was slower in high-SDI countries than in low-SDI countries, leading to a widening relative gap in total disease burden and a further shift toward low-SDI countries.

To further quantify these inequalities and their temporal evolution, we calculated the concentration index (CI). From 1990 to 2023, the CI for ASIR decreased from –0.168 to –0.277, with the absolute value increasing, indicating that the incidence burden became more concentrated in low-SDI countries. The CI for ASDR increased from –0.312 to –0.161, with the absolute value decreasing, suggesting that the death burden became less concentrated in low-SDI countries and began shifting toward high-SDI countries. The CI for ASDALYR increased from –0.362 to –0.310, with the absolute value also decreasing, indicating that the total disease burden remained concentrated in low-SDI countries, but its concentration lessened.

To further identify heterogeneity in disease burden trends across G20 countries, cluster analysis was performed. The results showed that G20 countries could be classified into four trend patterns, with China and India exhibiting similar trends to Germany and Japan (**Supplementary Figure 1**).

### 3.6 BAPC model projections

**Figure 9:**
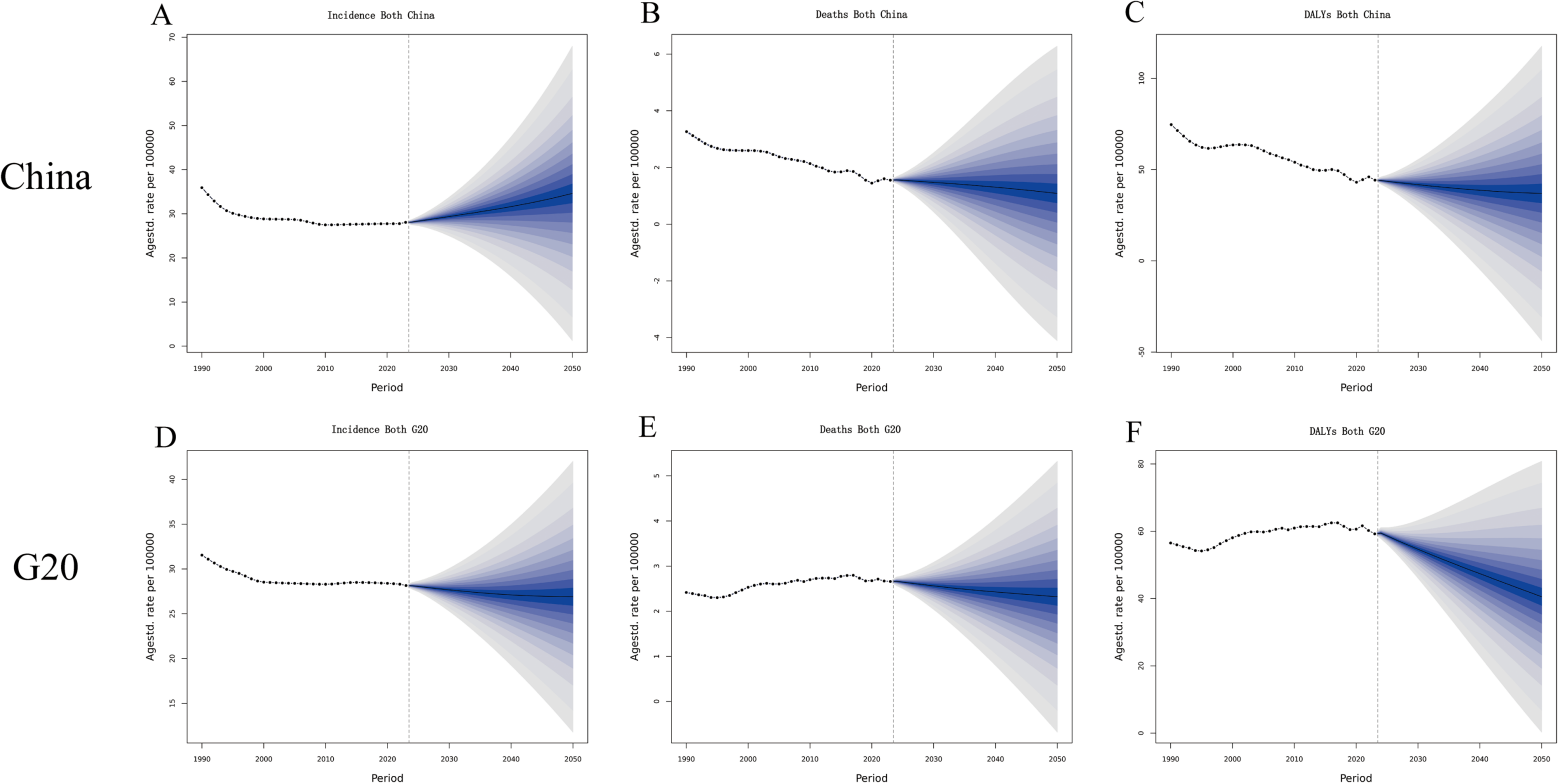
Time trends in ASIR, ASDR and ASDALYR for T2DM‑CKD from 1990 to 2023, including BAPC projections for 2024–2050. **(A)** China, incidence;**(B)** China, death;**(C)** China, DALYs;**(D)** G20 countries, incidence; **(E)** G20 countries, death; **(F)** G20 countries, DALYs. G20 in this analysis excludes China and the European Union to avoid double-counting. Shaded areas represent 95% uncertainty intervals (UI).

BAPC projections (**Figure 9**) show consistent upward trends with ARIMA forecasts for ASIR trends in China and G20 countries over 2024–2050. In China, the ASIR is projected to rise from 28.11 per 100, 000 (95% UI: 27.50–28.72) to 34.62 per 100, 000 (95% UI: 1.27–67.97), representing an increase of 23.16%. In contrast, the ASIR for G20 countries is projected to decrease from 28.12 per 100, 000 (95% UI: 27.75–28.50) to 26.90 per 100, 000 (95% UI: 11.78–42.02), a decline of 4.35%. Meanwhile, the ASDR and ASDALYR in both China and G20 countries are projected to show downward trends. In China, the ASDR is projected to decrease from 1.56 per 100, 000 (95% UI: 1.44–1.68) to 1.08 per 100, 000 (95% UI: 0 to 6.28); in G20 countries, from 2.66 per 100, 000 (95% UI: 2.57–2.76) to 2.32 per 100, 000 (95% UI: 0 to 5.32). Similarly, the ASDALYR in China is projected to decrease from 43.98 per 100, 000 (95% UI: 41.91–46.06) to 36.96 per 100, 000 (95% UI: 0 to 117.60); in G20 countries, from 59.45 per 100, 000 (95% UI: 57.80–61.10) to 40.55 per 100, 000 (95% UI: 0.30–80.81). In addition, BAPC projections for different age groups indicate that the ASIR, ASDR, and ASDALYR for T2DM-CKD across all age groups from 2024 to 2050 will generally align with the overall trends (**Supplementary Figure 4**; **Figure 5**).

### 3.7 ARIMA model forecast

**Figure 10:**
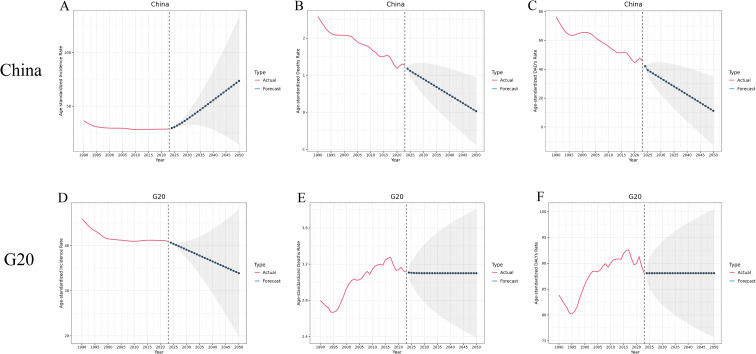
Time trends in ASIR, ASDR and ASDALYR for T2DM-CKD from 1990 to 2023, including ARIMA projections for 2024–2050. **(A)** China, incidence;**(B)** China, death;**(C)** China, DALYs;**(D)** G20 countries, incidence; **(E)** G20 countries, death; **(F)** G20 countries, DALYs. G20 in this analysis excludes China and the European Union to avoid double-counting. Shaded areas represent 95% confidence intervals (CI).

The ARIMA forecast (**Figure 10**) indicates that between 2024 and 2050, the ASDR and ASDALYR in China are expected to decline rapidly, dropping by 97.64% and 73.47%, respectively. In contrast, both ASDR and ASDALYR in G20 countries are projected to remain stable. Notably, the ASIR in China is projected to rise from 29.58 per 100, 000 (95% CI: 29.45–29.71) to 73.62 per 100, 000 (95% CI: 13.64–133.59), representing an increase of 148.88%. Meanwhile, the ASIR in G20 countries is projected to decline from 40.64 per 100, 000 (95% CI: 40.55–40.74) to 33.88 per 100, 000 (95% CI: 19.69–48.07)—a decrease of 16.64%.

Such dramatic projections highlight a known limitation: ARIMA forecasts are highly susceptible to over-extrapolation, as the model may erroneously extrapolate short-term fluctuations into long-term trends. Consequently, ARIMA results warrant cautious interpretation. In this study, ARIMA serves solely as a supplementary tool to evaluate overall trends and to corroborate the more robust BAPC projections.

## 4 Discussion

This study comprehensively analyzed the temporal trends in ASIR, ASDR and ASDALYR for T2DM-CKD in China and G20 countries from 1990 to 2023. Based on Joinpoint regression, the average annual percentage change (AAPC) was calculated for each indicator. China showed a decline in all three age-standardized rates from 1990 to 2023. The ASIR fell by 0.72% (95% CI: –0.76% to –0.67%), the ASDR fell by 2.02% (95% CI: –2.58% to –1.46%), and the ASDALYR fell by 1.45% (95% CI: –1.73% to –1.16%). China’s progress reflects not only rapid economic development but also continuous improvements in medical care. The sustained declines in ASDR and ASDALYR can be attributed to a series of healthcare system enhancements—including, but not limited to, expanded insurance coverage (18), improved access to dialysis (19), and the broader adoption of kidney-protective therapies (20).

Interestingly, we found that while Joinpoint analysis indicated a downward trend in China’s ASIR (**Figure 4**), the EAPC was positive (0.8, 95% CI: 0.46–1.14). The key lies in the fact that the decline in ASIR was not uniform; after a rapid initial decline, the rate gradually slowed, and there was even a slight increase during the longer period from 2010 to 2023 (APC = 0.13). This suggests that although prevention and control efforts for this disease have yielded notable success in recent years, substantial challenges remain—factors such as lifestyle changes and rising obesity rates make it difficult to sustain the downward trend in ASIR.

Joinpoint analysis identified several key turning points in China’s T2DM-CKD burden, reflecting how policy changes shaped disease outcomes. Between 1995 and 2002, the downward trend of ASDALYR slowed and briefly reversed (APC = 0.68%). During this time, healthcare system reforms were initiated in 1994 (18), the urban employee basic medical insurance system was established in 1998 with limited coverage (21), and the 2000 market-oriented reforms emphasized using drug sales to subsidize low medical service fees (22), increasing the personal financial burden of healthcare.

The resumption of a rapid decline from 2002 to 2013 (APC = –2.15%) was likely associated with the pilot of the New Rural Cooperative Medical Scheme in 2003 (1), the launch of the 2009 healthcare reform (18, 23), and a substantial increase in health insurance coverage. In 2012, the inclusion of uremia in the major disease insurance scheme significantly improved access to dialysis (24). From 2013 to 2017, the decline in ASDALYR and ASDR slowed (APC = –0.43%), possibly due to a transitional period of cost-control measures and drug price adjustments. The subsequent rapid decline from 2017 to 2020 (APC = –4.23%) may be attributed to the inclusion of kidney disease as a priority in the Medium- and Long-term Plan for the Prevention and Treatment of Chronic Diseases in China (25), as well as updates to the national drug reimbursement list that improved access to novel kidney-protective agents. Following a nadir in 2020, both ASDR and ASDALYR exhibited an upward trajectory through 2023. While this temporal alignment with the COVID-19 pandemic suggests a potential association (26), causality cannot be inferred from the current data; further validation incorporating post-pandemic observations is warranted. It is worth noting that G20 countries did not exhibit a similar trend to China’s between 2020 and 2023, suggesting that this change may be related to China’s unique response to the pandemic; however, the specific mechanisms require further study.

Although ASIR, ASDR, and ASDALYR showed a downward trend, the absolute number of cases in China continues to rise. A decomposition analysis reveals that China experienced the largest increase in the number of cases among G20 countries. Population aging is the primary factor driving the rise in the number of cases in China, accounting for 104.31%, 207.98% and 171.64% of the increases in incidence, death, and DALYs, respectively. Population growth is the second-largest contributor, accounting for 42.93%, 36.69%, and 30.68%, respectively. These findings are consistent with the progression of China’s population aging (27, 28). Furthermore, age-specific curves indicate that the age group with the highest DALYs in China has shifted from 75–79 years to 95 years and older. This phenomenon suggests an increase in the disease duration. China is facing two major challenges: population aging and the increasing duration of life lived with disease.

The divergence between declining age-standardized rates and rising absolute case numbers constitutes a fundamental ‘rate-number paradox’, encapsulating both clinical progress and escalating population-level challenges. On one hand, the reduction in ASDR and ASDALYS reflects improved individual outcomes, driven by a series of healthcare system enhancements—including expanded insurance coverage (18), improved access to dialysis (19), and the broader adoption of kidney-protective therapies (20). On the other hand, the absolute disease burden continues to surge, propelled primarily by population aging. This creates a structural imbalance between individual gains and collective burden: while individual patient prognosis improves, the rapidly expanding elderly demographic amplifies the total patient pool. Furthermore, the stagnant and post-2010 rebounding age-standardized incidence underscores a critical prevention-treatment gap, revealing a disproportionate allocation of policy resources toward end-stage management rather than early screening and lifestyle interventions. Given the irreversible nature of demographic aging, health resource planning needs to transcend rate-based metrics and explicitly account for the absolute scale of the elderly population.

Based on the contribution ratios, the absolute increase in the disease burden among G20 countries can be categorized into three main patterns: population aging-dominated (Japan, Mexico), a balance between population aging and population growth (India, Indonesia), and epidemiological changes-dominated (United States). Among these, the absolute increases in deaths and DALYs in the United States accounted for 39.27% and 27.00% of the total increase across G20 countries, respectively, both far exceeding those of other nations. Epidemiological changes contributed 68.04% and 61.56% of the absolute increases in deaths and DALYs, respectively. This may be attributed to poorly controlled risk factors (29), limited health insurance coverage (30), and wealth disparities (31).

After analyzing health inequalities across G20 countries, we found that the incidence burden (ASIR) became more concentrated in low-SDI countries (CI fell from –0.168 to –0.277). This may be due to rapid changes in lifestyle. While the prevalence of obesity and diabetes rose rapidly (32), health education and healthcare coverage lagged behind (33). At the same time, the death burden (ASDR) became less concentrated in low-SDI countries, with the CI rising from –0.312 to –0.161, suggesting a shift toward high-SDI countries. This likely reflects improved healthcare conditions in low-SDI countries, such as the widespread adoption of dialysis technology (34), whereas advanced medical technologies and more robust healthcare systems in high-SDI countries have extended patient survival. However, due to population aging and the large patient base, the absolute number of deaths remained high. Finally, the total burden (DALYs) remained concentrated in low-SDI countries, though the gap narrowed (CI from –0.362 to –0.31). This indicates that while advances in treatment technologies have extended patient survival, the number of new cases continued to surge, and the overall disease burden remained heavy.

Inequalities in prevention and treatment among G20 countries suggest that low-SDI countries need to strengthen primary prevention of this disease, while high-SDI countries need to further optimize long-term disease management to alleviate the burden of living with the disease over the long term. Therefore, as a developing country, China needs to enhance its capacity for prevention and response to this disease, while also learning from the long-term management experiences of high-SDI countries to reduce the disease burden.

The BAPC model, generally considered more reliable for long-term epidemiological forecasting, projects that China’s ASIR will rise by 23.2% to 34.62 per 100, 000 (95% UI: 1.27–67.97) by 2050, whereas ASDR and ASDALYR are anticipated to decline by 30.4% and 16.0%, respectively. The ARIMA model, which is prone to over-extrapolating transient fluctuations, produced implausibly extreme estimates (e.g., a 149% increase in ASIR) and is therefore de-emphasized. Given its capacity to integrate age-period-cohort effects through Bayesian smoothing, the BAPC model yields more conservative projections, which are generally viewed as more consistent with epidemiological patterns. Accordingly, the following policy implications are derived primarily from the BAPC projections.

Based on the preceding discussion, we propose the following recommendations.

For China, it is necessary to address the paradox between declining age-standardized rates and the absolute burden driven by population aging to mitigate the potential future rise in ASIR. First, we recommend increasing the screening frequency for urinary albumin-to-creatinine ratio (UACR) and estimated glomerular filtration rate (eGFR) among individuals aged 65 years and older with diabetes from annually to semiannually, while advancing the initiation of screening to the time of diabetes diagnosis. Second, patients with an eGFR <60 mL/min/1.73 m² should receive early intervention with kidney-protective agents such as SGLT2 inhibitors or ACEI/ARBs, supported by a multidisciplinary management model. Third, UACR screening should be incorporated into the annual free health examination package for diabetic patients over 65, reimbursement ratios for kidney-protective medications should be increased, and community-based health management for the elderly should be strengthened. Finally, resource planning for dialysis capacity, nephrology workforce, and community chronic disease management personnel should be based on the projected absolute size of the elderly population over the next 10–15 years.

Among G20 nations, strategies should be tailored to specific demographic drivers. For aging-dominated countries (e.g., Japan, Mexico), strategies similar to China’s are warranted: semiannual UACR/eGFR screening for diabetics over 65, prioritizing the inclusion of SGLT2 inhibitors in medical security schemes, and planning dialysis resources according to the absolute elderly population. For mixed-driver countries characterized by both population growth and aging (e.g., India, Indonesia), efforts should focus on strengthening primary prevention while expanding healthcare coverage; this should include listing essential dialysis services and kidney-protective drugs in national essential medicine lists and training primary care workers to perform timely UACR testing. For epidemiological change-dominated countries (e.g., the United States), the priority lies in comprehensive management of glycemia, blood pressure, and lipids, increasing insurance subsidies for low-income groups, and narrowing the accessibility gap for novel therapies such as SGLT2 inhibitors.

## 5 Limitations

This study has the following limitations. First, rather than raw survey data, the data are model-based estimates from multiple sources using DisMod-MR 2.1. Particularly in low-SDI countries with sparse data, the estimates may be subject to considerable uncertainty, which could affect the accuracy of international comparisons. Second, the decomposition analysis attributes the increase in disease burden to three components: population growth, population aging, and epidemiological changes. However, as a composite indicator, epidemiological changes cannot be further disaggregated to determine whether the increase is driven by changes in risk factors or improvements in diagnosis and treatment. Third, this study is based on macro-level data at the national level and precludes the inference of causal relationships at the individual level; therefore, the findings cannot be directly interpreted as changes in individual risk. Fourth, Joinpoint regression is sensitive to short-term fluctuations in recent data, which can affect the detection of inflection points. Consequently, recent upturns should be interpreted with caution, and extended follow-up is warranted to confirm whether they represent genuine trend reversals. Fifth, as both models are extrapolative and cannot incorporate unanticipated external shocks, the forecasts to 2050 serve as trend references rather than deterministic predictions. While the BAPC model provides stable and epidemiologically credible estimates (though potentially conservative), the ARIMA model demonstrates significantly expanding prediction intervals, underscoring its tendency toward over-extrapolation and the consequent escalation of uncertainty over the long term. Sixth, CI and SII can quantify the relationship between disease burden and SDI, but they cannot explain the specific mechanisms underlying inequality and are relatively sensitive to outliers. Seventh, the cluster analysis was limited to the G20 countries, resulting in a small sample size that may limit the stability of the classification results; therefore, this study treats the cluster analysis as an exploratory finding. Detailed results are presented in **Supplementary Figure 1**. Eighth, while the EAPC provides a summary statistic of overall change, it may be less informative for non-linear trends. Therefore, we emphasize that the EAPC should be interpreted in conjunction with the AAPC, which disaggregates trend dynamics across time segments, to achieve a comprehensive and accurate understanding of the temporal evolution of disease burden. Lastly, it is important to note that cross-country and temporal variations in coding standards, diagnostic practices, and data completeness may lead to discrepancies between GBD model-based estimates and the actual disease burden. Although the GBD team employs extensive data harmonization and garbage code redistribution algorithms to improve comparability, residual confounding cannot be entirely ruled out. Therefore, the findings should be interpreted with due caution.

## 6 Conclusion

From 1990 to 2023, China showed a general decline in the age-standardized incidence, death, and DALY rates (ASIR, ASDR, and ASDALYR) of T2DM-CKD. BAPC projections, regarded as more epidemiologically plausible than ARIMA, suggest a continued reduction in ASDR and ASDALYR alongside a projected persistent rise in ASIR. Nevertheless, driven largely by population aging, the absolute number of cases is expected to increase substantially, presenting an escalating challenge to China’s chronic disease burden.

Decomposition analysis categorized G20 nations into three distinct groups based on the drivers of absolute increases: aging-dominated (e.g., China), mixed (population growth and aging), and epidemiological change-dominated. Comparative analysis suggests that the incidence burden is increasingly concentrated in lower-SDI countries, while the death burden is less concentrated in higher-SDI regions—pointing to a critical disparity between prevention and treatment. Therefore, to address the dual challenge of rising incidence and insufficient therapeutic coverage, chronic disease management should be integrated into aging strategies, and a comprehensive, whole-cycle prevention framework should be established.

## Data Availability

Publicly available datasets were analyzed in this study. The data analyzed in this study are publicly available from the Institute for Health Metrics and Evaluation (IHME) Global Burden of Disease Study 2023. Data can be accessed through: Repository: Global Burden of Disease Study 2023 (GBD 2023) Direct link: https://vizhub.healthdata.org/gbd-results/Secondary link: https://ghdx.healthdata.org/No accession numbers are required. Users can freely query and download aggregated disease burden estimates by location, year, age group, sex, and cause. The datasets are not included in the article or **Supplementary Materials** due to size considerations.

## References

[1] GBD 2023 Disease and Injury and Risk Factor Collaborators . Burden of 375 diseases and injuries, risk-attributable burden of 88 risk factors, and healthy life expectancy in 204 countries and territories, including 660 subnational locations, 1990-2023: a systematic analysis for the Global Burden of Disease Study 2023. Lancet. (2025) 406:1873–922. doi: 10.1016/S0140-6736(25)01637-X 41092926 PMC12535840

[2] Fenta ET Eshetu HB Kebede N Bogale EK Zewdie A Kassie TD . Prevalence and predictors of chronic kidney disease among type 2 diabetic patients worldwide, systematic review and meta-analysis. Diabetol Metab Syndr. (2023) 15:245. doi: 10.1186/s13098-023-01202-x 38012781 PMC10683270

[3] Hu R Zhao Z Xie L Ma Z Wu W Li S . Global, regional, and national burden of chronic kidney disease due to diabetes mellitus type 2 from 1990 to 2021, with projections to 2036: a systematic analysis for the Global Burden of Disease Study 2021. Front Med (Lausanne). (2025) 12:1531811. doi: 10.3389/fmed.2025.1531811 40034386 PMC11872908

[4] Zhang Y Hou D Chai Z Li X Shan C Zhao Y . Long-term trends and future projections of the burden of diabetic nephropathy in China: a comprehensive analysis of GBD data from 1990 to 2036. Front Endocrinol (Lausanne). (2025) 16:1681689. doi: 10.3389/fendo.2025.1681689 41133223 PMC12541590

[5] Wang L Xu X Zhang M Hu C Zhang X Li C . Prevalence of chronic kidney disease in China: Results from the Sixth China Chronic Disease and Risk Factor Surveillance. JAMA Intern Med. (2023) 183:298–310. doi: 10.1001/jamainternmed.2022.6817 36804760 PMC9941971

[6] Dieleman JL Cowling K Agyepong IA Alkenbrack S Bollyky TJ Bump JB . The G20 and development assistance for health: historical trends and crucial questions to inform a new era. Lancet. (2019) 394:173–83. doi: 10.1016/S0140-6736(19)31333-9 31257126

[7] GBD 2021 Risk Factors Collaborators . Global burden and strength of evidence for 88 risk factors in 204 countries and 811 subnational locations, 1990-2021: a systematic analysis for the Global Burden of Disease Study 2021. Lancet. (2024) 403:2162–203. doi: 10.1016/S0140-6736(24)00933-4 38762324 PMC11120204

[8] GBD 2023 Chronic Kidney Disease Collaborators . Global, regional, and national burden of chronic kidney disease in adults, 1990-2023, and its attributable risk factors: a systematic analysis for the Global Burden of Disease Study 2023. Lancet. (2025) 406:2461–82. doi: 10.1016/S0140-6736(25)01853-7 41213283

[9] Cao Y Chen H Liu H Wu H Gao W . Global, regional, and national temporal trends in incidence for type 2 diabetes mellitus related chronic kidney disease from 1992 to 2021. Diabetes Metab J. (2025) 49:848–61. doi: 10.4093/dmj.2024.0593 40068620 PMC12270589

[10] Xie D Ma T Cui H Li J Zhang A Sheng Z . Global burden and influencing factors of chronic kidney disease due to type 2 diabetes in adults aged 20-59 years, 1990-2019. Sci Rep. (2023) 13:20234. doi: 10.1038/s41598-023-47091-y 37981642 PMC10658077

[11] Das Gupta P . A general method of decomposing a difference between two rates into several components. Demography. (1978) 15:99–112. doi: 10.2307/2060493 631402

[12] Xu Q Qiao Z Kan Y Wan B Qiu X Yang Y . Global, regional, and national burden of depression, 1990-2021: a decomposition and age-period-cohort analysis with projection to 2040. J Affect Disord. (2025) 391:120018. doi: 10.1016/j.jad.2025.120018 40782921

[13] Wagstaff A Paci P van Doorslaer E . On the measurement of inequalities in health. Soc Sci Med. (1991) 33:545–57. doi: 10.1016/0277-9536(91)90212-u 1962226

[14] Knoll M Furkel J Debus J Abdollahi A Karch A Stock C . An R package for an integrated evaluation of statistical approaches to cancer incidence projection. BMC Med Res Methodol. (2020) 20:257. doi: 10.1186/s12874-020-01133-5 33059585 PMC7559591

[15] Gascoigne C Riebler A Smith T . Smooth predictions for age-period-cohort models: a comparison between splines and random process. BMC Med Res Methodol. (2025) 25:177. doi: 10.1186/s12874-025-02629-8 40721740 PMC12306036

[16] Schaffer AL Dobbins TA Pearson SA . Interrupted time series analysis using autoregressive integrated moving average (ARIMA) models: a guide for evaluating large-scale health interventions. BMC Med Res Methodol. (2021) 21:58. doi: 10.1186/s12874-021-01235-8 33752604 PMC7986567

[17] Deng Q Wang S Lyu J . Comparative effectiveness analysis of univariate time-series forecasting models for disease mortality rates in the global burden of disease database: a case study of global hypertensive heart disease among women of childbearing age. Front Public Health. (2025) 13:1681569. doi: 10.3389/fpubh.2025.1681569 41393012 PMC12696747

[18] Yip W Fu H Chen AT Zhai T Jian W Xu R . 10 years of health-care reform in China: progress and gaps in Universal Health Coverage. Lancet. (2019) 394:1192–204. doi: 10.1016/S0140-6736(19)32136-1 31571602

[19] Lin L Zai X . Assessing the impact of public insurance on healthcare utilization and mortality: A nationwide study in China. SSM Popul Health. (2024) 25:101615. doi: 10.1016/j.ssmph.2024.101615 38322784 PMC10844660

[20] Li C Guo S Huo J Gao Y Yan Y Zhao Z . Real-world national trends and socio-economic factors preference of sodium-glucose cotransporter-2 inhibitors and glucagon-like peptide-1 receptor agonists in China. Front Endocrinol (Lausanne). (2022) 13:987081. doi: 10.3389/fendo.2022.987081 36277697 PMC9585197

[21] Xu L Wang Y Collins CD Tang S . Urban health insurance reform and coverage in China using data from National Health Services Surveys in 1998 and 2003. BMC Health Serv Res. (2007) 7:37. doi: 10.1186/1472-6963-7-37 17335584 PMC1828155

[22] General Office of the State Council . Notice of the general office of the state council on transmitting the guiding opinions of the state council body reform office and other departments on the reform of the urban medical and health system (Guobanfa [2000] no. 16). Beijing, China: Gov China (2000). Available online at: http://www.gov.cn/gongbao/content/2000/content_60046.htm/ (Accessed June 6, 2026).

[23] Central Committee of the Communist Party of China, State Council of the People's Republic of China . Opinions of the central committee of the communist party of China and the state council on deepening the reform of medicine and health system. Beijing, China: Gov China (2009). Available online at: https://www.gov.cn/gongbao/content/2009/content_1284372.htm/ (Accessed June 6, 2026).

[24] National Development and Reform Commission, Ministry of Health, Ministry of Finance, et al . Guiding opinions on launching critical illness insurance for urban and rural residents(Fagai shehui [2012] no. 2605). Beijing, China: NDRC China (2012). Available online at: https://www.ndrc.gov.cn/xx_gk/zcfb/tz/201208/t20120830_964475.html/ (Accessed June 6, 2026).

[25] General Office of the State Council . Circular of the general office of the state council on issuing the medium- and long-term plan for the prevention and treatment of chronic diseases in China (2017-2025) (Guobanfa [2017] no. 12). Beijing, China: Gov China (2017). Available online at: https://www.gov.cn/zhengce/content/2017-02/14/content_5167886.htm/ (Accessed June 6, 2026).

[26] Li Y Tong X Jiang M Wu Z Duan Y Zhang L . Chronic disease and infection in China: lessons from the covid-19 pandemic. BMJ. (2024) 387:q2000. doi: 10.1136/bmj.q2000 39424310 PMC12036597

[27] OECD . Elderly population (indicator). Paris, France: OECD Data (2025). Available online at: https://www.oecd.org/en/data/indicators/elderly-population.html/ (Accessed June 6, 2026).

[28] Chen X Giles J Yao Y Yip W Meng Q Berkman L . The path to healthy ageing in China: a Peking University-Lancet Commission. Lancet. (2022) 400:1967–2006. doi: 10.1016/S0140-6736(22)01546-X 36423650 PMC9801271

[29] Corpodean F Kachmar M Yang S Heymsfield SB Katzmarzyk PT Schauer PR . Association of obesity severity with cardiometabolic and renal disease burden in the United States. Obesity. (2026) 34:323–8. doi: 10.1002/oby.70099 41250827 PMC12834209

[30] McCoy RG Vandergrift JL Gray B . Patient and physician factors driving the gaps in use of drugs with cardiovascular and kidney benefits by medicare beneficiaries with type 2 diabetes treated by endocrinologists, nephrologists, and cardiologists: Population-based cohort study. Diabetes Res Clin Pract. (2025) 221:112039. doi: 10.1016/j.diabres.2025.112039 39923965

[31] Lin Z Wang W Xie S Jiang B Zhang X Xu Y . Social risk profiles and diabetic kidney disease: prevalence and mortality in US adults. BMC Public Health. (2025) 25:3065. doi: 10.1186/s12889-025-24272-0 40993757 PMC12462275

[32] NCD Risk Factor Collaboration (NCD-RisC) . Worldwide trends in underweight and obesity from 1990 to 2022: a pooled analysis of 3663 population-representative studies with 222 million children, adolescents, and adults. Lancet. (2024) 403:1027–50. doi: 10.1016/S0140-6736(23)02750-2 38432237 PMC7615769

[33] Teerawattananon Y Chavarina KK Phannajit J Sutawong J Yongphiphatwong N Tang SCW . The Access to Dialysis in Low- and Middle-Income Countries Commission: lessons for universal health coverage. Nat Med. (2025) 31:19–21. doi: 10.1038/s41591-024-03448-y 39843854

[34] Nkunu V Tungsanga S Diongole HM Sarki A Arruebo S Caskey FJ . Landscape of kidney replacement therapy provision in low- and lower-middle income countries: A multinational study from the ISN-GKHA. PloS Glob Public Health. (2024) 4:e0003979. doi: 10.1371/journal.pgph.0003979 39621612 PMC11611141

