# Supplementary material for: Burden and trends of chronic kidney disease due to type 2 diabetes mellitus in China and G20 countries, 1990–2023: a comparative analysis
Source: Front Endocrinol (Lausanne). 2026 Jun 10;17:1853478. doi: 10.3389/fendo.2026.1853478 (PMC13290614; doi:10.3389/fendo.2026.1853478)

**Supplementary Figure 1.** Cluster analysis of G20 countries based on average annual percentage change (AAPC) in ASIR, ASDR, and ASDALYR for T2DM‑CKD, 1990–2023. Countries were classified into four trend patterns: significant increase, moderate increase, moderate decrease, and significant decrease.


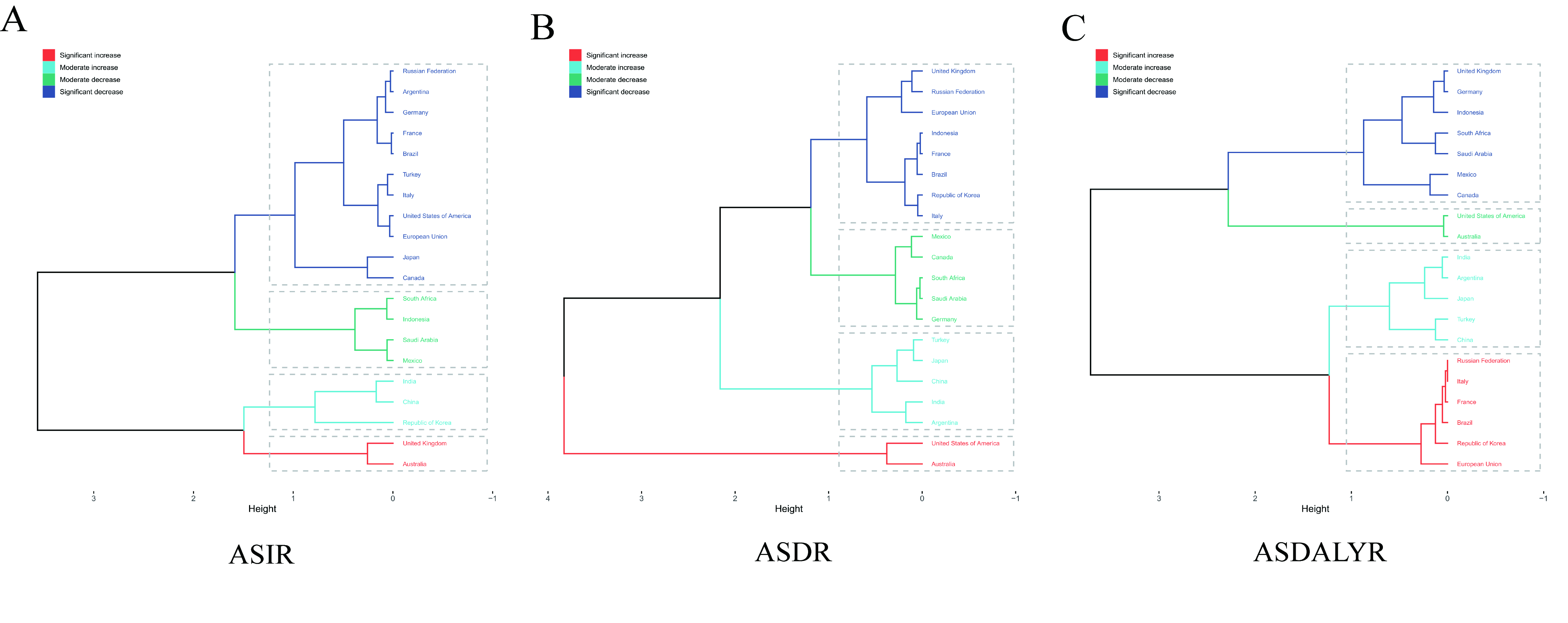

Supplement: Supplementary file 1 [file DataSheet1.docx]
