# Supplementary material for: Burden and trends of chronic kidney disease due to type 2 diabetes mellitus in China and G20 countries, 1990–2023: a comparative analysis
Source: Front Endocrinol (Lausanne). 2026 Jun 10;17:1853478. doi: 10.3389/fendo.2026.1853478 (PMC13290614; doi:10.3389/fendo.2026.1853478)

**Supplementary Figure 2.** Bivariate analysis of T2DM CKD by sex and age in China and G20 countries. The figure displays sex and age stratified case numbers (population pyramids) and sex specific age standardized rate trends for incidence, death, and DALYs over 1990–2023. (A) China, ASIR; (B) China, ASDR; (C) China, ASDALYR; (D) G20 countries, ASIR; (E) G20 countries, ASDR; (F) G20 countries, ASDALYR.
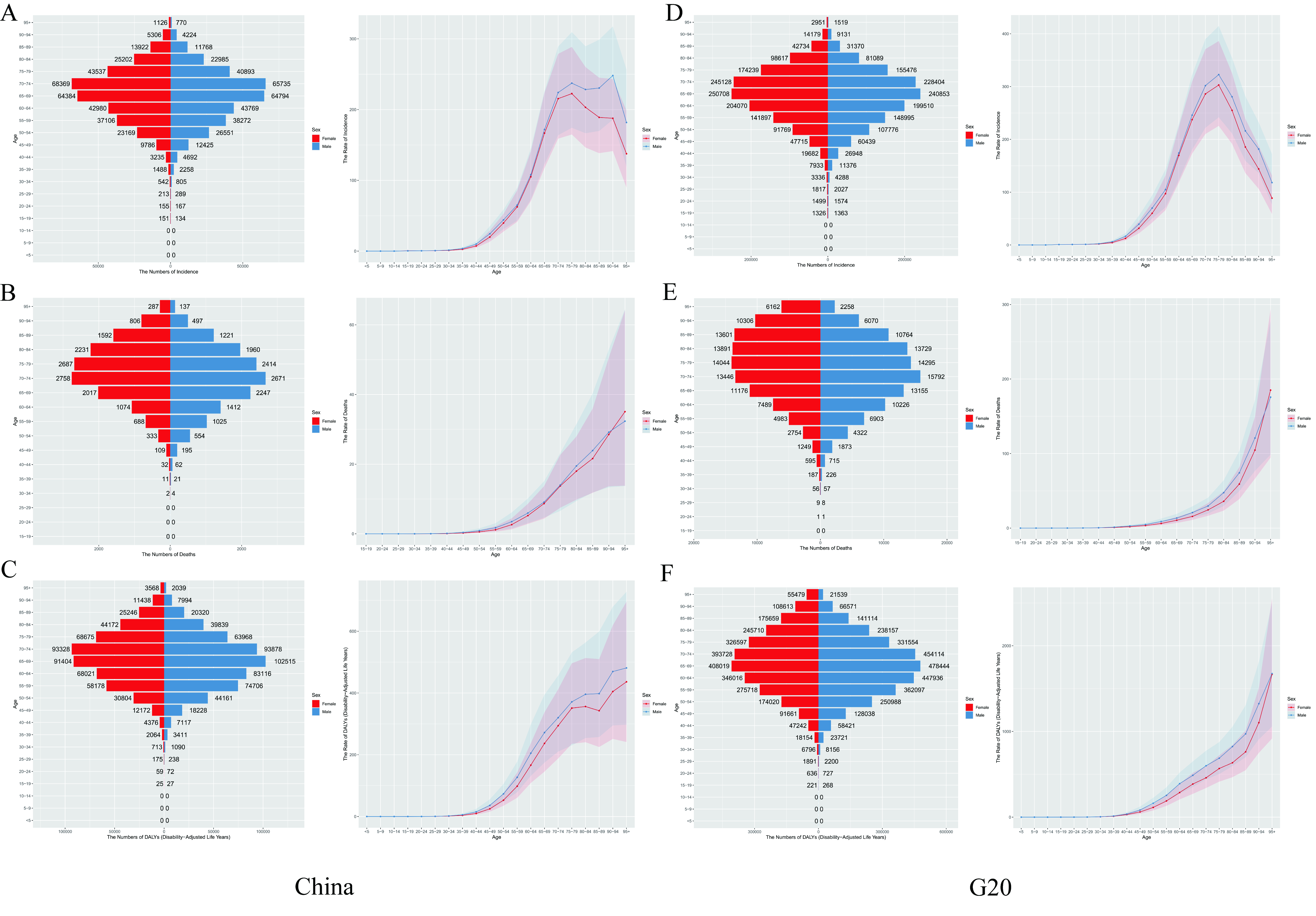

Supplement: Supplementary file 2 [file DataSheet2.docx]
