# Supplementary material for: Burden and trends of chronic kidney disease due to type 2 diabetes mellitus in China and G20 countries, 1990–2023: a comparative analysis
Source: Front Endocrinol (Lausanne). 2026 Jun 10;17:1853478. doi: 10.3389/fendo.2026.1853478 (PMC13290614; doi:10.3389/fendo.2026.1853478)

**Supplementary Figure 3.** Absolute changes in ASDR and ASIR for T2DM CKD in G20 countries, 1990–2023. Map colors represent ΔASR = ASR_2023 − ASR_1990. Blue indicates ΔASR < 0; red/orange indicate ΔASR > 0.


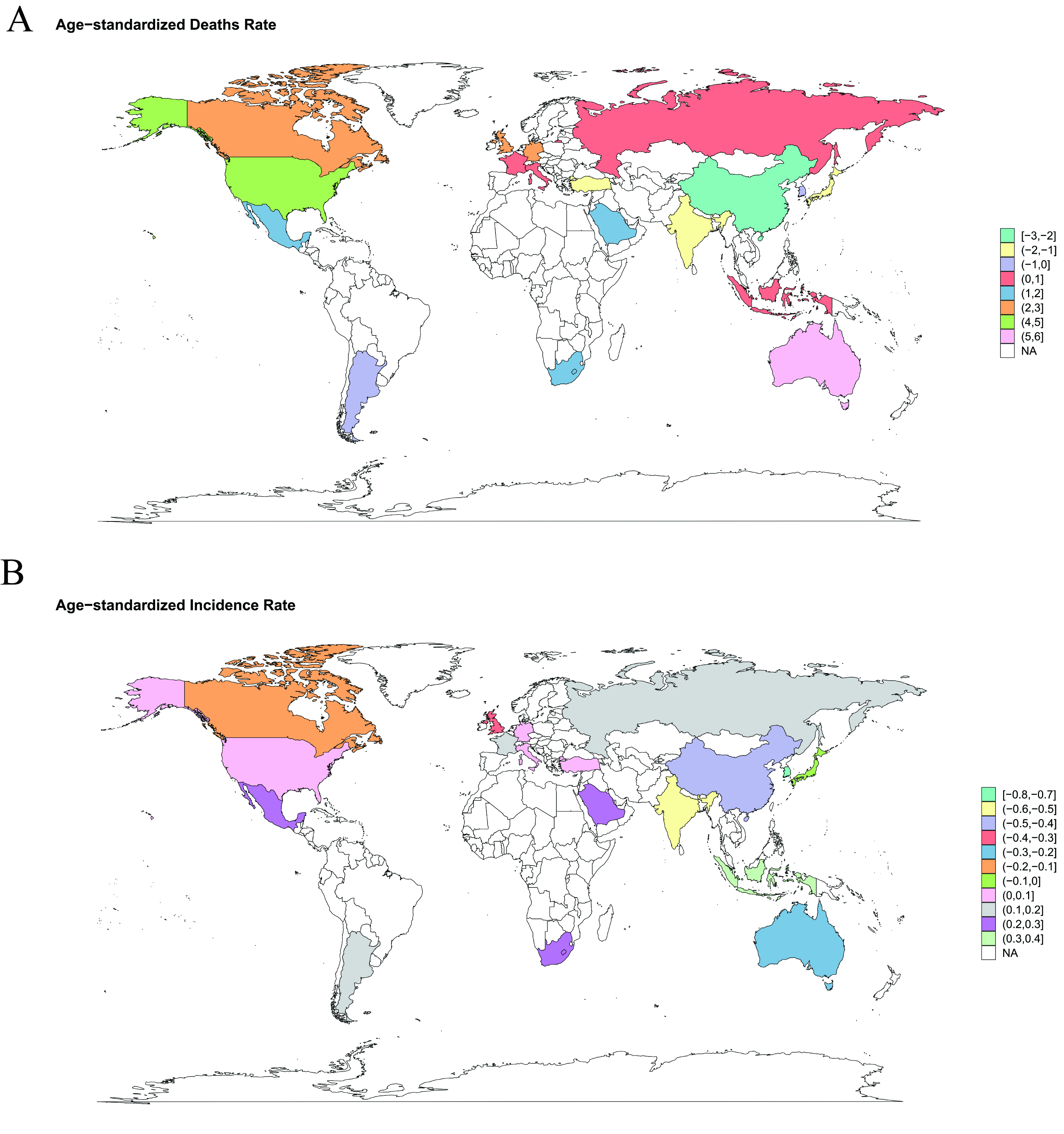

Supplement: Supplementary file 3 [file DataSheet3.docx]
