# Supplementary material for: Burden and trends of chronic kidney disease due to type 2 diabetes mellitus in China and G20 countries, 1990–2023: a comparative analysis
Source: Front Endocrinol (Lausanne). 2026 Jun 10;17:1853478. doi: 10.3389/fendo.2026.1853478 (PMC13290614; doi:10.3389/fendo.2026.1853478)

**Supplementary Figures 4 and Figure 5.** Bayesian age period cohort (BAPC) projections of age standardized incidence, death, and DALY rates (ASIR, ASDR, ASDALYR) for T2DM CKD by age group in China and G20 countries, 2024–2050. (Figure 4) China; (Figure 5) G20.


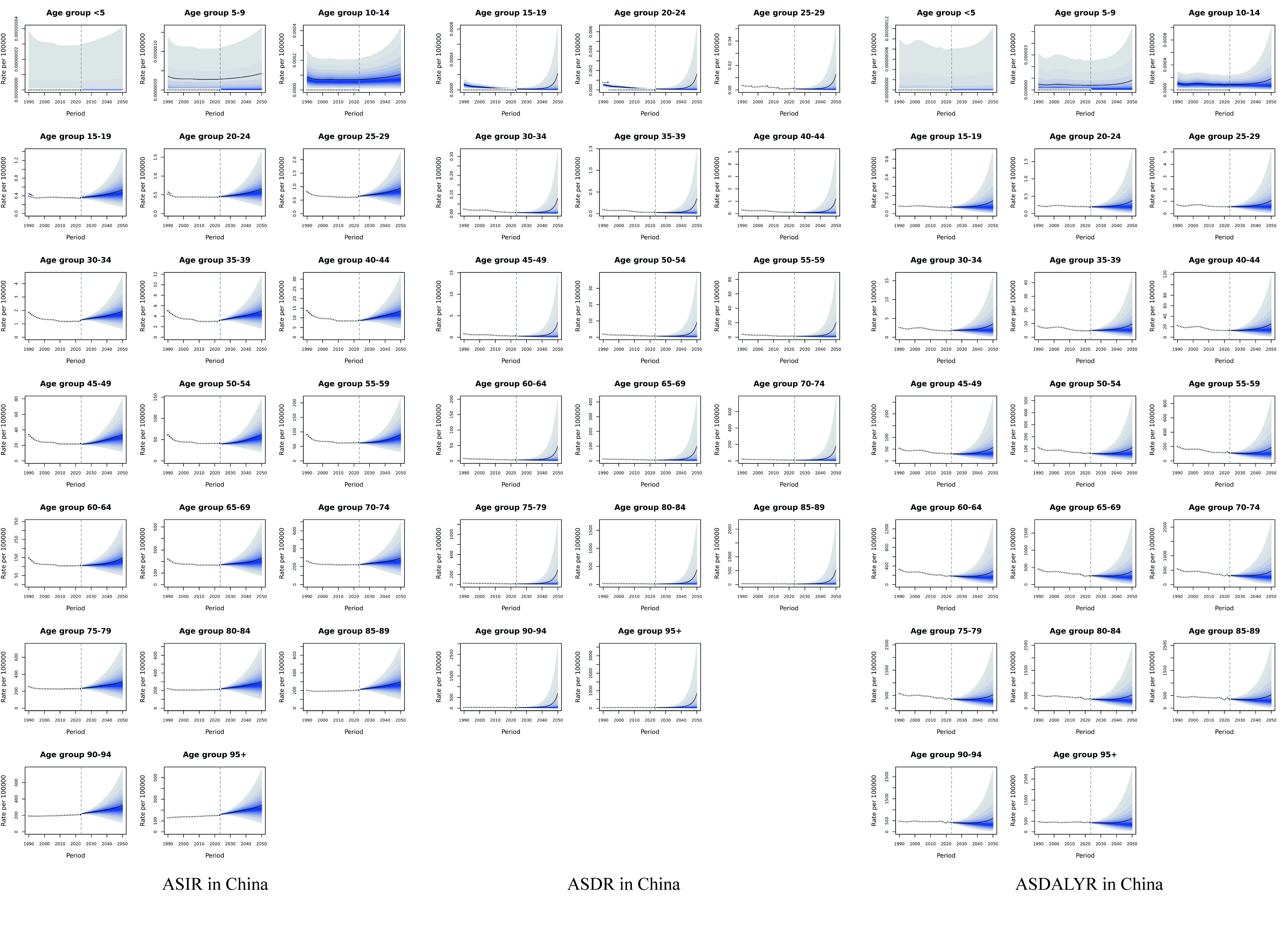


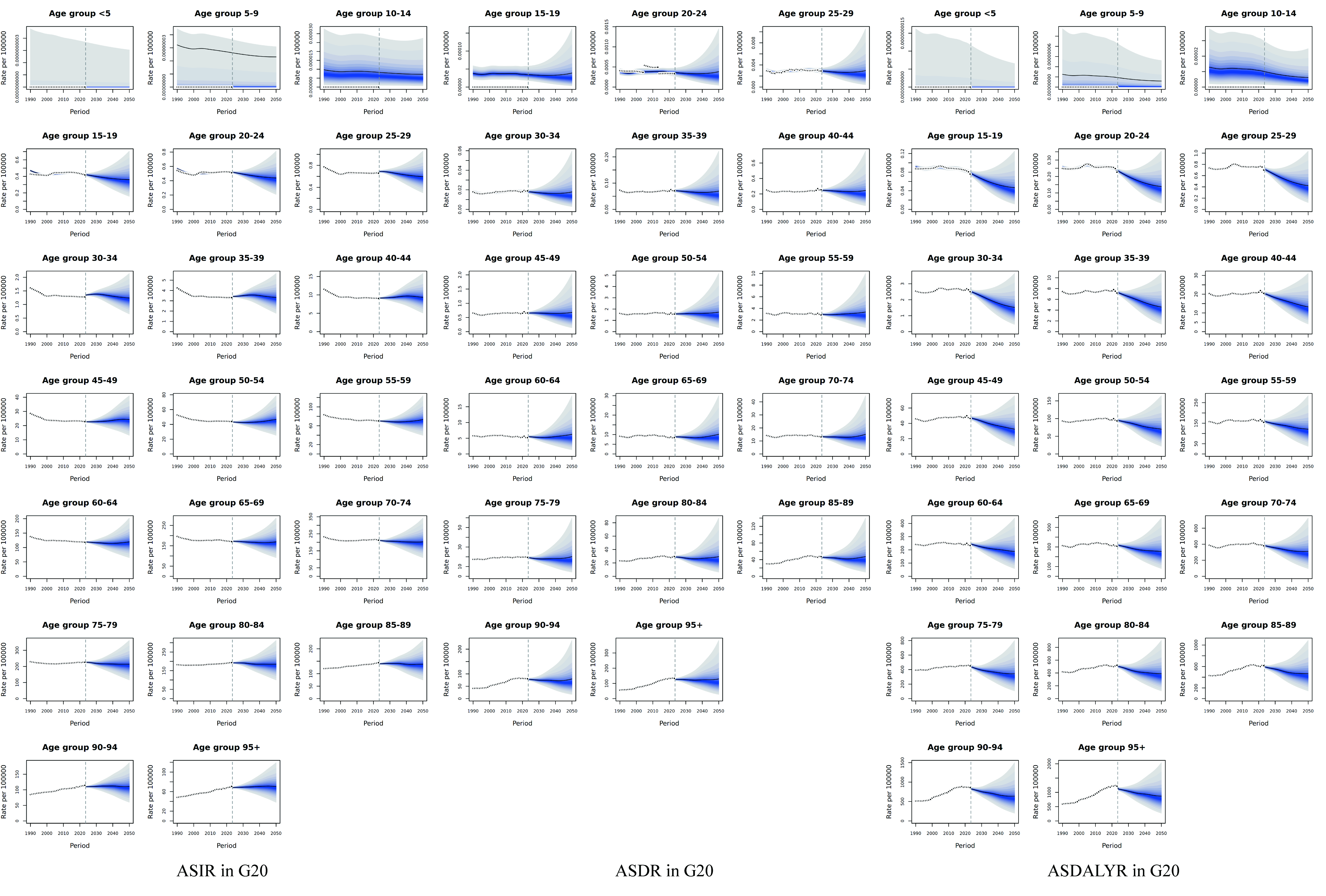

Supplement: Supplementary file 4 [file DataSheet4.docx]
