# Supplementary material for: Burden and trends of chronic kidney disease due to type 2 diabetes mellitus in China and G20 countries, 1990–2023: a comparative analysis
Source: Front Endocrinol (Lausanne). 2026 Jun 10;17:1853478. doi: 10.3389/fendo.2026.1853478 (PMC13290614; doi:10.3389/fendo.2026.1853478)
Supplement: Supplementary file 5 [file DataSheet5.docx]

**Supplementary Table 1. Joinpoint regression analysis of temporal trends in age-standardized rates of type 2 diabetes-related kidney disease burden in China, 1990-2023**

| **Indicator** | **Sex** | **Period** | **APC (95% CI)** | **P-value** |
| --- | --- | --- | --- | --- |
| DALYs | Both | 1990-1995 | -3.6341(-4.3419--2.9211) | <0.05 |
|  |  | 1995-2002 | 0.6767(0.2080-1.1477) | <0.05 |
|  |  | 2002-2013 | -2.1533(-2.3316--1.9747) | <0.05 |
|  |  | 2013-2017 | -0.4321(-1.5455-0.6939) | ns |
|  |  | 2017-2020 | -4.2296(-6.3845--2.0251) | <0.05 |
|  |  | 2020-2023 | 1.4449(0.3105-2.5922) | <0.05 |
|  | Female | 1990-1995 | -3.877(-4.3977--3.3534) | <0.05 |
|  |  | 1995-2002 | 0.8396(0.4676-1.2129) | <0.05 |
|  |  | 2002-2013 | -2.1564(-2.3028--2.0098) | <0.05 |
|  |  | 2013-2017 | -0.6557(-1.5764-0.2737) | ns |
|  |  | 2017-2020 | -4.7431(-6.4894--2.9642) | <0.05 |
|  |  | 2020-2023 | 2.0404(1.0957-2.9938) | <0.05 |
|  | Male | 1990-1995 | -3.4918(-4.4834--2.4900) | <0.05 |
|  |  | 1995-2002 | 0.4595(-0.1864-1.1096) | ns |
|  |  | 2002-2014 | -2.1359(-2.3231--1.9485) | <0.05 |
|  |  | 2014-2017 | 0.3348(-2.6332-3.3933) | ns |
|  |  | 2017-2020 | -3.9589(-6.3810--1.4741) | <0.05 |
|  |  | 2020-2023 | 1.0238(-0.3308-2.3968) | ns |
| Deaths | Both | 1990-1995 | -3.8466(-5.1109--2.5655) | <0.05 |
|  |  | 1995-2003 | -0.42(-1.0954-0.2601) | ns |
|  |  | 2003-2014 | -2.6559(-2.9671--2.3437) | <0.05 |
|  |  | 2014-2017 | 0.0223(-3.9767-4.1879) | ns |
|  |  | 2017-2020 | -7.727(-11.2865--4.0247) | <0.05 |
|  |  | 2020-2023 | 3.1426(1.1550-5.1693) | <0.05 |
|  | Female | 1990-1995 | -3.7314(-4.7335--2.7187) | <0.05 |
|  |  | 1995-2002 | -0.0799(-0.7933-0.6386) | ns |
|  |  | 2002-2014 | -2.4133(-2.6522--2.1737) | <0.05 |
|  |  | 2014-2017 | -0.1141(-3.4774-3.3663) | ns |
|  |  | 2017-2020 | -8.2768(-11.2497--5.2043) | <0.05 |
|  |  | 2020-2023 | 3.3999(1.6871-5.1415) | <0.05 |
|  | Male | 1990-1994 | -4.4152(-6.9729--1.7871) | <0.05 |
|  |  | 1994-2007 | -1.2735(-1.6555--0.8902) | <0.05 |
|  |  | 2007-2014 | -3.5873(-4.4740--2.6925) | <0.05 |
|  |  | 2014-2017 | 0.5894(-5.1469-6.6726) | ns |
|  |  | 2017-2020 | -7.2953(-11.9865--2.3541) | <0.05 |
|  |  | 2020-2023 | 2.8498(0.2201-5.5484) | <0.05 |
| Incidence | Both | 1990-1994 | -3.8068(-3.9881--3.6253) | <0.05 |
|  |  | 1990-1994 | -3.8915(-4.0009--3.7818) | <0.05 |
|  |  | 1994-1999 | -1.0395(-1.2144--0.8643) | <0.05 |
|  |  | 1999-2005 | -0.0623(-0.1840-0.0596) | ns |
|  |  | 2005-2010 | -0.8533(-1.0237--0.6826) | <0.05 |
|  |  | 2010-2023 | 0.1288(0.1007-0.1569) | <0.05 |
|  | Female | 1990-1994 | -3.8915(-4.0009--3.7818) | <0.05 |
|  |  | 1994-1999 | -1.4091(-1.5149--1.3031) | <0.05 |
|  |  | 1999-2005 | -0.0853(-0.1585--0.0120) | <0.05 |
|  |  | 2005-2010 | -0.9568(-1.0588--0.8547) | <0.05 |
|  |  | 2010-2023 | 0.0756(0.0586-0.0926) | <0.05 |
|  | Male | 1990-1994 | -3.7367(-3.8248--3.6484) | <0.05 |
|  |  | 1994-1998 | -0.8062(-0.9395--0.6727) | <0.05 |
|  |  | 1998-2005 | -0.1286(-0.1730--0.0842) | <0.05 |
|  |  | 2005-2010 | -0.715(-0.7969--0.6330) | <0.05 |
|  |  | 2010-2021 | 0.1241(0.1042-0.1439) | <0.05 |
|  |  | 2021-2023 | 0.7436(0.4868-1.0012) | <0.05 |

**Supplementary Table 2. Joinpoint regression analysis of temporal trends in age-standardized rates of type 2 diabetes-related kidney disease burden in G20 countries, 1990-2023**

| **Indicator** | **Sex** | **Period** | **APC (95% CI)** | **P-value** |
| --- | --- | --- | --- | --- |
| DALYs | Both | 1990-1995 | -0.9768(-1.4024--0.5495) | <0.05 |
|  |  | 1995-2002 | 1.3917(1.0784-1.7060) | <0.05 |
|  |  | 2002-2017 | 0.3101(0.2328-0.3874) | <0.05 |
|  |  | 2017-2023 | -0.6408(-0.9086--0.3724) | <0.05 |
|  | Female | 1990-1995 | -1.0432(-1.3424--0.7432) | <0.05 |
|  |  | 1995-2001 | 2.0405(1.7461-2.3358) | <0.05 |
|  |  | 2001-2013 | 0.3302(0.2503-0.4103) | <0.05 |
|  |  | 2013-2016 | 0.9631(-0.1556-2.0944) | ns |
|  |  | 2016-2019 | -1.112(-2.2991-0.0895) | ns |
|  |  | 2019-2023 | 0.0004(-0.3974-0.3997) | ns |
|  | Male | 1990-1995 | -1.1015(-1.7513--0.4474) | <0.05 |
|  |  | 1995-2002 | 0.9927(0.5350-1.4525) | <0.05 |
|  |  | 2002-2017 | 0.2513(0.1426-0.3601) | <0.05 |
|  |  | 2017-2023 | -0.778(-1.1351--0.4196) | <0.05 |
| Deaths | Both | 1990-1996 | -0.7722(-1.2272--0.3151) | <0.05 |
|  |  | 1996-2001 | 2.3343(1.5244-3.1506) | <0.05 |
|  |  | 2001-2016 | 0.5795(0.4755-0.6836) | <0.05 |
|  |  | 2016-2023 | -0.6939(-0.9561--0.4310) | <0.05 |
|  | Female | 1990-1995 | -0.9959(-1.4905--0.4988) | <0.05 |
|  |  | 1995-2001 | 2.5774(2.0917-3.0654) | <0.05 |
|  |  | 2001-2017 | 0.6442(0.5688-0.7196) | <0.05 |
|  |  | 2017-2020 | -1.7624(-3.4267--0.0695) | <0.05 |
|  |  | 2020-2023 | 0.6598(-0.2487-1.5767) | ns |
|  | Male | 1990-1996 | -1.1124(-1.5983--0.6241) | <0.05 |
|  |  | 1996-2001 | 1.6539(0.7487-2.5672) | <0.05 |
|  |  | 2001-2016 | 0.4623(0.3540-0.5709) | <0.05 |
|  |  | 2016-2023 | -0.7818(-1.0643--0.4984) | <0.05 |
| Incidence | Both | 1990-1993 | -1.3685(-1.5070--1.2298) | <0.05 |
|  |  | 1993-1999 | -0.9353(-0.9955--0.8751) | <0.05 |
|  |  | 1999-2002 | -0.2625(-0.5249-0.0007) | ns |
|  |  | 2002-2010 | -0.1195(-0.1546--0.0844) | <0.05 |
|  |  | 2010-2016 | 0.1187(0.0612-0.1764) | <0.05 |
|  |  | 2016-2023 | -0.0781(-0.1129--0.0432) | <0.05 |
|  | Female | 1990-1993 | -1.332(-1.4129--1.2510) | <0.05 |
|  |  | 1993-1999 | -0.8377(-0.8726--0.8029) | <0.05 |
|  |  | 1999-2009 | -0.1839(-0.1978--0.1700) | <0.05 |
|  |  | 2009-2016 | 0.0769(0.0513-0.1025) | <0.05 |
|  |  | 2016-2021 | -0.0893(-0.1374--0.0411) | <0.05 |
|  |  | 2021-2023 | -0.4028(-0.5560--0.2494) | <0.05 |
|  | Male | 1990-1993 | -1.4483(-1.5708--1.3257) | <0.05 |
|  |  | 1993-1999 | -1.0818(-1.1348--1.0288) | <0.05 |
|  |  | 1999-2002 | -0.2759(-0.5077--0.0436) | <0.05 |
|  |  | 2002-2010 | -0.1158(-0.1462--0.0853) | <0.05 |
|  |  | 2010-2015 | 0.1319(0.0612-0.2027) | <0.05 |
|  |  | 2015-2023 | -0.0011(-0.0260-0.0238) | ns |

*Note: APC, annual percent change; CI, confidence interval; ns, not significant (P ≥ 0.05). The "G20" excludes both China (to allow for separate analysis) and the European Union (to prevent duplication of member states).*
